# Phenanthridine–pyrene conjugates as fluorescent probes for DNA/RNA and an inactive mutant of dipeptidyl peptidase enzyme

**DOI:** 10.3762/bjoc.19.40

**Published:** 2023-04-26

**Authors:** Josipa Matić, Tana Tandarić, Marijana Radić Stojković, Filip Šupljika, Zrinka Karačić, Ana Tomašić Paić, Lucija Horvat, Robert Vianello, Lidija-Marija Tumir

**Affiliations:** 1 Laboratory for Biomolecular Interactions and Spectroscopy, Division of Organic Chemistry and Biochemistry, Ruđer Bošković Institute, Bijenička cesta 54, 10000 Zagreb, Croatia; 2 Laboratory for the Computational Design and Synthesis of Functional Materials, Division of Organic Chemistry and Biochemistry, Ruđer Bošković Institute, Bijenička cesta 54, 10000 Zagreb, Croatia; 3 Laboratory for Physical Chemistry and Corrosion, Department of Chemistry and Biochemistry, Faculty of Food Technology and Biotechnology, University of Zagreb, Croatia; 4 Laboratory for Protein Biochemistry and Molecular Modelling, Division of Organic Chemistry and Biochemistry, Ruđer Bošković Institute, Bijenička cesta 54, 10000 Zagreb, Croatia; 5 Laboratory for Molecular Plant Biology and Biotechnology, Division of Molecular Biology, Ruđer Bošković Institute, Bijenička cesta 54, 10000 Zagreb, Croatia

**Keywords:** dipeptidyl peptidase enzyme, excimer, molecular dynamics simulations, phenanthridine, polynucleotide, pyrene

## Abstract

Two novel conjugate molecules were designed: pyrene and phenanthridine-amino acid units with a different linker length between the aromatic fragments. Molecular modelling combined with spectrophotometric experiments revealed that in neutral and acidic buffered water solutions conjugates predominantly exist in intramolecularly stacked conformations because of the π–π stacking interaction between pyrene and phenanthridine moieties. The investigated systems exhibited a pH-dependent excimer formation that is significantly red-shifted relative to the pyrene and phenanthridine fluorescence. While the conjugate with a short linker showed negligible spectrophotometric changes due to the polynucleotide addition, the conjugate with a longer and more flexible linker exhibited a micromolar and submicromolar binding affinity for ds-polynucleotides and inactivated a mutant of dipeptidyl peptidase enzyme E451A. Confocal microscopy revealed that the conjugate with the longer linker entered the HeLa cell membranes and blue fluorescence was visualized as the dye accumulated in the cell membrane.

## Introduction

The design of small molecules that can selectively bind and discriminate different biomolecular structures (polynucleotides vs proteins, DNA or RNA, single or double-stranded polynucleotides, particular base composition…) and signalize binding by specific spectroscopic response is of great importance [1–2].

Pyrene derivatives are among the earliest known fluorescent probes for biomolecules. These chromophores are often used due to their high extinction coefficient and long emission lifetime (>100 ns) [3]. Their large aromatic hydrophobic surface allows the intercalation between DNA/RNA base pairs and binding within the minor groove. Pyrenes are also prominent protein probes that can monitor protein conformational changes because of pyrene sensitivity to the polarity of its surroundings. Similar as pyrenes, phenanthridines are also used as fluorescent probes, and their characteristics may be altered by various substituents appended to the aromatic core. The formation of excimers by two or more pyrenes is well known in the literature [4]. Excimers are formed when pyrene moieties form supramolecular complexes by intermolecular or intramolecular π–π interactions, causing a significant shift of single pyrene bands to longer wavelengths. Pyrenes are often used as a sensor part of receptor molecules, so their excimer bands switch on/off to signalize complex formation (interaction with a biomolecule, cyclodextrine, metal cation, etc.) or change of receptor conformation [5–6]. Employment of pyrene as a biosensor is complicated due to its large aromatic surface's hydrophobicity and fluorescence sensitivity to oxygen. Therefore, modifications of the pyrene unit as well as combinations of pyrene with other aromatic fluorophores could improve the properties [7]. Recently, Takaishi et al. reported chiral exciplex dyes having pyrenyl, perylenyl, and 4-(dimethylamino)phenyl groups incorporated in their structure, which showed circularly polarized luminescence (CPL). The exciplex (excited heterodimer) formed intramolecularly proved to be conformationally rigid and consequently was not sensitive to solvent or temperature [8]. Further, pyrenoimidazole-fused phenanthridines have been reported recently, developed as fluorescence emitters for optoelectronic applications [9]. These compounds have a very large aromatic surface suitable to form self-assembled supramolecular structures by intermolecular π–π interactions and showed excimer fluorescence in thin film and in the solid state. Kawai et al. reported exciplex formation between pyrene and guanine in polar solvents, including water. The exciplex was formed by the intramolecular interaction of guanine and pyrene, linked by a flexible methylene chain [10].

Our lab has conducted considerable research on phenanthridine derivatives, and earlier research has demonstrated that two phenanthridine units can also combine to produce an excimer, which is identifiable by a certain fluorescence band [11–12].

Some of the pyrenes and phenanthridines exhibited meaningful biological activity. Several pyrene-guanidiniocarbonylpyrrole derivatives have been found to exhibit the affinity for ds-DNA that is strongly pH-dependent, and the flexibility of the linker can alter that [13]. Further, pyrene-guanidiniocarbonylpyrrole discriminated DNA and RNA by different spectroscopic (induced circular dichroism signal and fluorescent signal) responses [14]. Also, we recently reported a pyrene–quinoline conjugate molecule that formed an exciplex [15], and conjugates formed of pyrene and an amino acid-fluorescent nucleobase derivative qAN1, differing in length and flexibility between fluorophores [16]. Due to pre-organization, both conjugates strongly interacted with ds-DNA/RNA grooves with similar affinity but opposite fluorescence response. Compounds that consisted of pyrrole-guanidine attached to larger aryl moieties (pyrene and phenanthridine) bind to the human DPP III enzyme [17]. Pyrene–cyanine conjugates connected with a rigid triazole-peptide linker were designed and synthesized in our group and showed a strong pyrene emission change upon binding to proteins, and a cyanine fluorescence that was selective for polynucleotides. Moreover, the FRET pair of chromophores was activated upon binding to biomolecules [18].

**Scheme 1:**
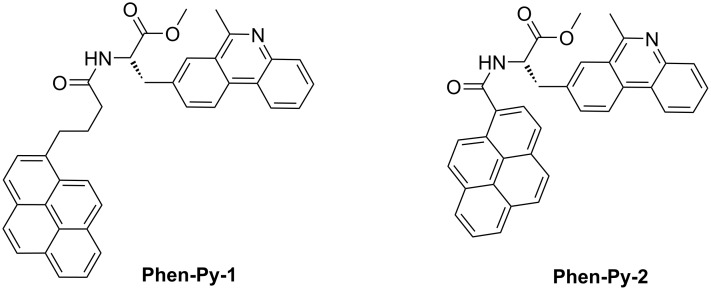
Novel pyrene–phenanthridine conjugates **Phen-Py-1** (longer, flexible linker) and **Phen-Py-2** (shorter, rigid linker).

**Scheme 2:**
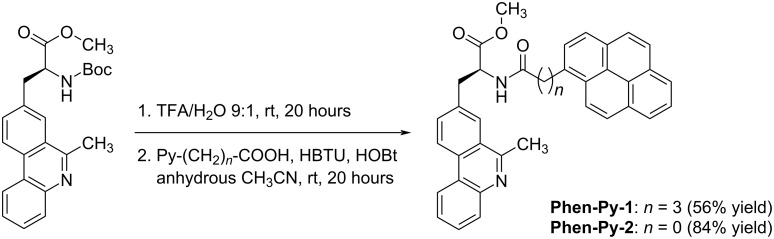
Synthesis of **Phen-Py-1** and **Phen-Py-2** by amide formation; Reagents and conditions: 1. TFA–H_2_O mixture (9:1, v/v; 2 mL), rt, 20 hours. 2. Anhydrous acetonitrile, HBTU, HOBt, Et_3_N, rt, 20 hours.

Continuing our previous work, two phenanthridine–pyrene conjugates **Phen-Py-1** and **Phen-Py-2** (Scheme 1), differing only in the linker length between the aromatic units, have been prepared by condensation of two different pyrenecarboxylic acids with phenanthridine-labelled amino acid (Scheme 2).

The influence of the linker length on the molecule flexibility, intramolecular conformation, spectroscopic properties, and polynucleotide binding affinity has been investigated by UV–vis, fluorescence and CD spectroscopy and molecular modeling. Further, binding of **Phen-Py-1** to human dipeptidyl peptidase III enzyme was investigated by fluorescence spectroscopy and microcalorimetric measurements.

## Results and Discussion

### Synthesis

The phenanthridine derivative of the amino acid alanine (**Phen-AA**) has been prepared according to the procedure described earlier [19]. **Phen-AA** was deprotected under acidic conditions (TFA–H_2_O) at room temperature for 20 hours. The amide coupling reaction was performed in anhydrous acetonitrile with pyrenecarboxylic acid in the presence of triethylamine (TEA), *N*,*N*,*N’*,*N*′-tetramethyl-*O*-(1*H*-benzotriazol-1-yl)uronium hexafluorophosphate (HBTU) as the coupling reagent, and 1-hydroxybenzotriazole (HOBT) as a coupling additive to give the products **Phen-Py-1** and **Phen-Py-2** (Scheme 2) in 56% and 84% yield, respectively. Compounds are stable in the refrigerator for more than six months; however, after one year partial decomposition of sample **Phen-Py-2** was observed.

### Spectroscopic characterization of **Phen-Py-1** and **Phen-Py-2** in aqueous solution

#### UV–vis spectra

Studied compounds **Phen-Py-1** and **Phen-Py-2** were moderately soluble in DMSO (up to *c* = 1 × 10^−3^ mol dm^−3^) and their stock solutions were stable during a few months. All measurements were recorded in the Na cacodylate buffer (*I_c_* = 0.05 mol dm^−3^) both at pH 5.0 and pH 7.0 for comparison, since the phenanthridine heterocyclic nitrogen becomes protonated in weakly acidic conditions (pH 5) [20–21]. The volume ratio of DMSO was less than 1% in all measurements. The absorbance of aqueous solutions for compounds **Phen-Py-1** and **Phen-Py-2** was proportional to their concentrations up to *c* = 1–2 × 10^−5^ mol dm^−3^. In contrast to the Lambert–Beer law, a decrease of UV–vis spectra upon heating up to 90 °C and a baseline increase indicated intermolecular stacking and aggregation of compounds, which was more pronounced for **Phen-Py-2**. Spectroscopic characterization data are given in the Table 1 and Supporting Information File 1, Figure S1.

A linker between chromophores enabled intramolecular contacts between the phenanthridine and pyrene chromophore. This stacking interaction minimized the surface area that was exposed to water. As a result, mutual shielding of chromophores and coulombic interaction between induced dipoles could cause hypochromism and consequent decrease of the ε value [22–24], although this decrease was not an accurate measure of the shielding degree [25–27].

To determine the hypochromic effect (% H) at a single wavelength, the UV–vis absorption of the examined compounds was compared with the absorption of reference compounds (Scheme S2, Supporting Information File 1) that possessed the same chromophores [20,28–29]. Therefore, the UV–vis absorption of **Phen-Py-1** and **Phen-Py-2** was compared with the sum of the absorption of **Phen-AA** [11,19] (comprising phenanthridine unit, Scheme S2, Supporting Information File 1) and the absorption of 1-pyrenebutyric acid **PBA** (containing a pyrene unit, Scheme S2, Supporting Information File 1). A noticeable hypochromic effect (% H) was observed for both **Phen-Py-1** and **Phen-Py-2** (Table 1). The hypochromic effect was more potent at pH 5 (55% and 53% for **Phen-Py-1** and **Phen-Py-2**, respectively) than at pH 7 (35% and 48% for **Phen-Py-1** and **Phen-Py-2**, respectively). The phenanthridine nitrogen was protonated under weakly acidic conditions, which made the phenanthridinium moiety relatively electron deficient compared to the electron-rich pyrene moiety that favored intramolecular stacking. Molecular dynamics simulations additionally supported the pronounced hypochromic effect (chapter Computational analysis).

#### Fluorescence spectra

Fluorescence emission of **Phen-Py-1** and **Phen-Py-2** measured at pH 5 and pH 7 (cacodylate buffer, *I_c_* = 0.05 mol dm^−3^) was linearly dependent on the concentration up to 4 × 10^−6^ mol dm^−3^. (Figure S3, Supporting Information File 1). Emission quantum yields in acidic and neutral water solutions for **Phen-Py-1** are given in Table 1.

**Table 1:** Electronic absorption data and quantum yields of **Phen-Py-1** and **Phen-Py-2** (sodium cacodylate/HCl buffer, *I_c_* = 0.05 mol dm^−3^, pH 5 or pH 7) and reference compounds **Phen-AA** and **Pyr**.

	pH 5				pH 7			
	λ_max_ (nm)	ε (mmol^−1^ cm^2^)	H^a^ (%)	^c^Φ_f_ (%)	λ_max_ (nm)	ε (mmol^−1^ cm^2^)	H^b^ (%)	Φ_f_^c^ (%)

**Phen-Py-1**	276 329 343	19387 10994 11140	55%	8.49	277 331 346	35310 18451 19756	35%	12.74
**Phen-Py-2**	276 348	20059 9741	53%	^d^	278 277 333 350	27895 28154 13018 13697	48%	^d^

^a^H (hypochromic effect, %)_276 nm_ = 100 × {[ε_276 nm_ (**Phen-AA**) + ε_276 nm_ (**PBA**)] − ε_276 nm_ (**Phen-Py-1** or **Phen-Py-2**)_276 nm_ }/ [ε_276 nm_ (**Phen-AA**) + ε_276 nm_ (**PBA**)]; ε_276 nm_ (**Phen-AA**) = 11.35 mol^−1^ cm^2^; ε_276 nm_ (**PBA**) = 31.65 mol^−1^ cm^2^; ε_276 nm_ (**Phen-Py-1**) = 19.39 mol^−1^ cm^2^; ε_276 nm_ (**Phen-Py-2**) = 20.06 mol^−1^ cm^2^; (pH 5.0). ^b^H (hypochromic effect, %)_277 nm_ = 100 × {[ε_277 nm_ (**Phen-AA**) + ε_277 nm_ (**PBA**)] − ε_277 nm_ (**Phen-Py-1** or **Phen-Py-2**)_277 nm_ }/ [ε_277 nm_ (**Phen-AA**) + ε_277 nm_ (**PBA**)]; ε_277 nm_ (**Phen-AA**) = 9.20 mol^−1^ cm^2^; ε_277 nm_ (**PBA**) = 44.91 mol^−1^ cm^2^; ε_277 nm_ (**Phen-Py-1**) = 35.31 mol^−1^ cm^2^; ε_277 nm_ (**Phen-Py-2**) = 28.15 mol^−1^ cm^2^ (pH 7.0). ^c^The absolute fluorescence quantum yield was determined by integrating sphere SC-30, Edinburgh Inst., for argon purged solutions. ^d^Not determined.

**Figure 1:**
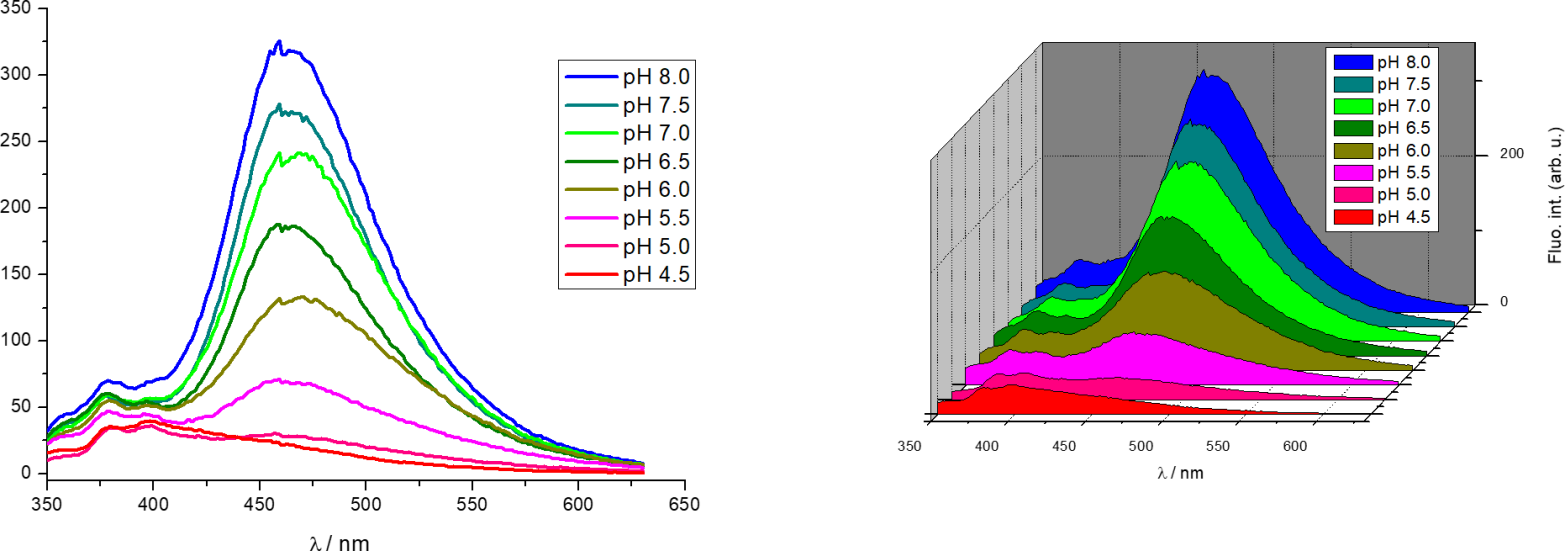
2D (left) and 3D (right) representation of fluorescence emission spectra of **Phen-Py-1** (*c* = 2 × 10^−6^ mol dm^−3^) at different pH values (Na cacodylate, HCl, *I_c_* = 0.05 mol dm^−3^ at 25 °C (λ_exc_ = 280 nm)).

Excitation spectra of conjugates **Phen-Py-1** and **Phen-Py-2** were in good agreement with their UV–vis spectra. Phenanthridine–pyrene conjugate **Phen-Py-1** exhibited excimer formation characterized by a new fluorescence emission band at 475 nm, which is significantly red-shifted compared to either fluorescent emission of single phenanthridine (λ_max_ = 400 nm) or pyrene (λ_max_ = 378 and 400 nm) molecule (Supporting Information File 1, Figure S2, left pH 5.0; right pH 7.0). **Phen-Py-2** also showed a shoulder at 480 nm (besides the main emission signal at 400 nm) which could be attributed to the excimer formation (Figure S2, Supporting Information File 1). A similar new red-shifted emission band was noticed for other exciplex examples: pyrene–guanine [10] pyrene–quinolone [15], pyrene–perylene [8]. For **Phen-Py-1**, the excimer formation was observed both upon excitation at 280 nm and 350 nm and it was found to be pH-dependent (Figure 1). Further, the excimer signal was observed in water, but not in methanol (Figure S5, Supporting Information File 1), due to a lower dielectric constant and a lower polarity that influenced intramolecular stacking. To the best of our knowledge, this is the first reported phenanthridine–pyrene excimer in solution.

Molecular dynamics calculations (see chapter Computational analysis) and hypochromism observed from UV–vis spectra pointed towards stronger intramolecular stacking interactions at weakly acidic conditions (pH 5.0) compared to pH 7 for both compounds. Also, the pyrene–phenanthridine π-overlap was more pronounced for **Phen-Py-2**. On the other hand, the excimer signal (475 nm) of **Phen-Py-1** was significantly stronger at weakly basic and neutral conditions at which intramolecular stacking was less pronounced. At acidic conditions, the monomer signal (400 nm) was dominant (Figure 1).

The emission of the excimer did not exclusively depend on the degree of overlapping of chromophores. Earlier theoretical examinations favored a symmetrical sandwich configuration as optimal for the largest exciton splitting, while other reports suggested a favored orientation with one aromatic moiety displaced (ca 1.4 Å) from the other to minimize van der Waals repulsion [30]. Redistribution of charges at acidic pH (protonated phenanthridine nitrogen) compared to neutral pH (unprotonated phenanthridine nitrogen) caused more efficient stacking and the hypochromic effect. However, the energy transfer between chromophores and resulting excimer fluorescence was increased at neutral and basic conditions. The excimer fluorescence was obviously sensitive to small changes in the mutual orientation of chromophores.

### Computational analysis

To examine conformational features of **Phen-Py-1** and **Phen-Py-2**, and inspect whether their intrinsic dynamics in aqueous solution play a role in determining their ability to form stacked aggregates and excimers, we performed molecular dynamics (MD) simulations of different protonation forms of **Phen-Py-1** and **Phen-Py-2** placed in explicit water solvation, and analyzed structural preferences in the obtained trajectories.

**Figure 2:**
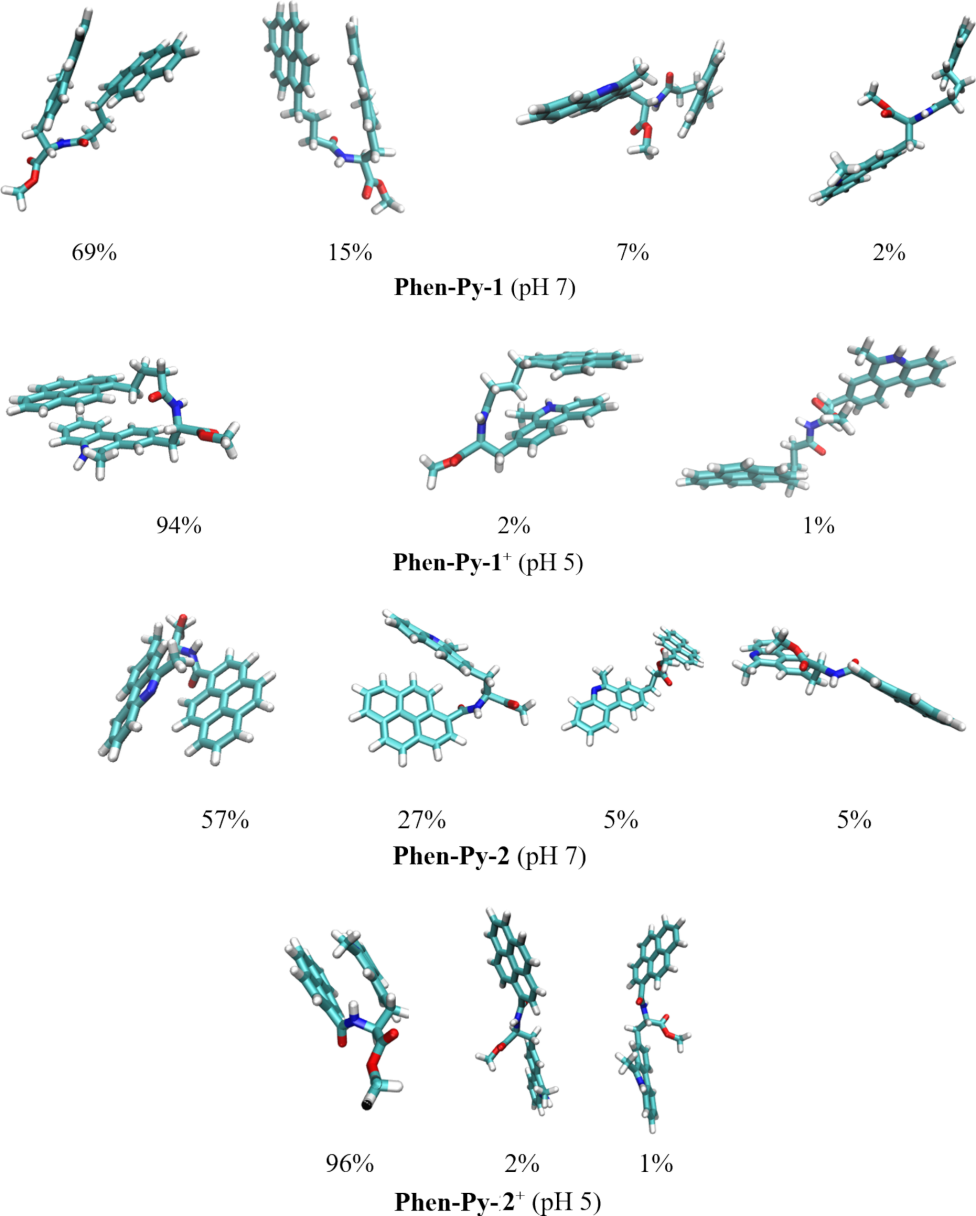
Most representative structures of the conjugates **Phen-Py-1** and **Phen-Py-2** at different pH conditions and their overall populations during MD simulations. These were identified after the clustering analysis of the corresponding MD trajectories.

In setting up our simulations, we prepared the geometries of unionized **Phen-Py-1** and **Phen-Py-2** and their monocations, protonated at the phenanthridine nitrogen atom. These structures correspond to pH values of pH 7 and pH 5, respectively, in accordance with experiments conducted here. Taking the experimental p*K*_a_ value of the isolated phenanthridine, p*K*_a_ = 4.65 [31] our calculated p*K*_a_ of methylphenanthridine is p*K*_a_ = 6.3. We assumed that this value would not change much in the prepared conjugates, which confirms that both **Phen-Py-1** and **Phen-Py-2** are monoprotonated at pH 5. We submitted all four systems to molecular dynamics simulations and performed clustering analysis on the obtained structures. The most representative geometries that account for most of the population of each system are presented in Figure 2.

The results showed that both conjugates, irrespective of their protonation state, prefer stacked conformations, with favorable π–π interactions among aromatic fragments. Interestingly, this also holds even for a formally more rigid **Phen-Py-2**, as it turned out that the ethyl chain possesses enough flexibility to enable the intramolecular contacts. Still, one observes that folded structures are more frequent in monoprotonated derivatives **Phen-Py-1^+^** and **Phen-Py-2^+^**, where the stacking π–π interactions are further promoted by favorable cation–π interactions. In other words, in **Phen-Py-1^+^** and **Phen-Py-2^+^**, stacked structures account for around 96% and 98% of population, respectively, while in unionized **Phen-Py-1** and **Phen-Py-2** these cluster around 84% in both cases (Figure 2). This is further supported by inspecting the evolution of distances between the centers of mass among aromatic units (Figure S22, Supporting Information File 1), which are found around 5.5 Å for both conjugates under acidic conditions, at the same time exceeding 8 Å (**Phen-Py-1**) and 6 Å (**Phen-Py-2**) under neutral conditions. All of this convincingly indicates that monoprotonated analogues are less available for both the intermolecular interactions among systems in solution and the subsequent formation of intermolecular excimers.

Further, although interaction of pyrene and phenanthridine is necessary for the appearance of excimer band it seems that a higher degree of aromatic surfaces overlapping and cation–π interactions also yield excimer fluorescence quenching [30,32–33]. This conclusion is strongly in line with experimental insight reported here and helps in explaining the observed excimer fluorescence quenching with a decrease in the solution pH value as well as with the stronger excimer fluorescence of **Phen-Py-1** compared to **Phen-Py-2**.

We calculated the energies of the excited states responsible for the experimental UV–vis spectra in Supporting Information File 1, Figure S25 corresponding to isolated conjugates to test the validity of the assumption that the systems **Phen-Py-1** and **Phen-Py-2** are well represented in the aqueous solution with the predominant structures shown in Figure 2. In order to do this, we selected the most prevalent structure in each system in Figure 2, optimized the geometry using the M06-2X/6-31+G(d) method, and then did TD-DFT calculations at the same level of theory. Solvent effects were modeled using the SMD implicit water solvation. The obtained vertical transitions corresponding to absorption maxima at pH 7.0 are 260 and 372 nm (**Phen-Py-1**), and 270 and 397 nm (**Phen-Py-2**). At pH 5.0 vertical transitions are 265 and 357 nm (**Phen-Py-1^+^**), and 266 and 366 nm (**Phen-Py-2^+^**). They are discovered to be in very excellent agreement with the experimental findings shown in Table 1, which supports the computational strategy used here and demonstrates the reliability of the clustering analysis. This result is further supported by the fact that using the same methodology, the isolated phenanthridine showed an absorption maximum of 256 nm at pH 7.0, which is in perfect agreement with the experimental value of 248 nm [34] and well-matched with 250 nm reported here for **Phen-AA** (Table 1).

### Interactions of **Phen-Py-1** and **Phen-Py-2** with biomolecules

Conjugates **Phen-Py-1** and **Phen-Py-2** were examined for DNA/RNA binding affinity and eventual preference for different polynucleotide structures. For example, the B-helical structure had a well-defined minor groove which is suitable for minor groove binding, while A-helical structure is favorable for intercalation and/or major groove binding. *Calf thymus* DNA (*ct-*DNA, 58% AT and 42% GC base pairs) and poly rA–poly rU (RNA) were chosen as models for a classical B-helical and A-helical structure, respectively [35]. Unlike **Phen-Py-2**, compound **Phen-Py-1** showed notable spectroscopic response upon binding, thus additional experiments of **Phen-Py-1** with synthetic DNA polynucleotides [23], poly(dAdT)_2_ and poly(dGdC)_2_, and enzyme dipeptidyl peptidase III (E451, inactive DPP III mutant) were performed. To explore the potential of dyes as new fluorescent probes, we have studied cellular uptake and intracellular distribution of **Phen-Py-1** in HeLa cells by TCS SP8 X confocal microscopy. The results showed that the dye entered the HeLa cell membranes fast, and after 1 hour of incubation at 1 µM concentration, blue fluorescence was visualized as the dye accumulated in the cell membrane (Figure S26, Supporting Information File 1). The compound showed not to be toxic to the HeLa cells as no visible damage was detected.

### Interactions of **Phen-Py-1** and **Phen-Py-2** with *ds*-polynucleotides and enzyme dipeptidyl peptidase III in an aqueous medium

Interactions of **Phen-Py-1** and **Phen-Py-2** with DNA and RNA were studied by fluorimetric titrations, thermal melting experiments, and CD titrations. Excimer bands showed photobleaching; therefore, for the spectrophotometric titrations, buffered solutions of compounds were prepared 24 hours before, to ensure stabile compound spectra. Fluorimetric titrations of both compounds with DNA/RNA showed only negligible and/or linear fluorescence change at weakly acidic conditions (protonated form of the conjugate molecule, pH 5, Figures S9–S12, Supporting Information File 1). Further, **Phen-Py-1** and **Phen-Py-2** showed negligible and/or linear fluorescence change upon the addition of *ct-*DNA also at pH 7 (Figures S13 and S14, Supporting Information File 1), while for **Phen-Py-1**, more notable fluorescence changes at pH 7 (neutral form) were observed. Therefore, all further studies were performed at pH 7.

### Thermal melting studies

Binding of small molecules to double stranded DNA or RNA polynucleotides usually affect the double helix stability. This stabilization or destabilization was revealed as a change of the polynucleotide’s melting temperature (Δ*T*_m_ value) which was the difference of the *T*_m_ value of free polynucleotide and the *T*_m_ value of polynucleotide–small molecule complex [36]. Thermal melting experiments showed that conjugates **Phen-Py-1** and **Phen-Py-2** had only a negligible effect on the double helix stability (below 0.5 °C), both for DNA and RNA (Table S1, Figures S6–S8, Supporting Information File 1).

### Spectrophotometric titrations

**Figure 3:**
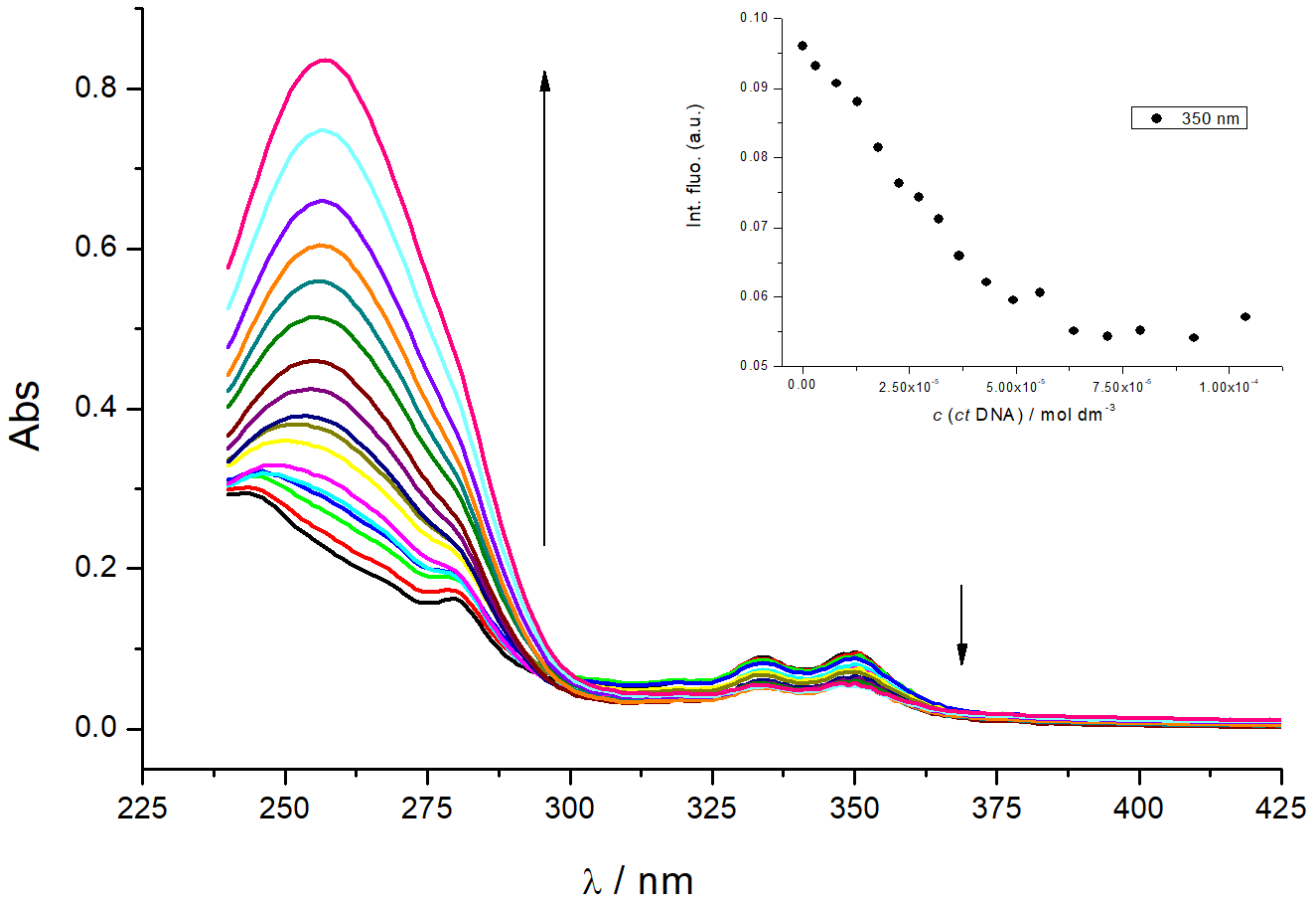
UV–vis titration of **Phen-Py-1** with *ct*-DNA,; changes in the UV–vis spectra of **Phen-Py-1** at λ = 350 nm. (*c* (**Phen-Py-1)** = 1 × 10^−5^ mol dm^−3^, sodium cacodylate buffer, pH 7, *I_c_* = 0.05 mol dm^−3^).

The addition of *ct*-DNA to a **Phen-Py-1** solution resulted in a hypochromic effect in the region 325–365 nm (Figure 3) without a shift of absorption maxima. Systematic deviation of the isosbestic points around 300 nm revealed the coexistence of more than two spectroscopically active species and more than one dominant binding mode.

**Figure 4:**
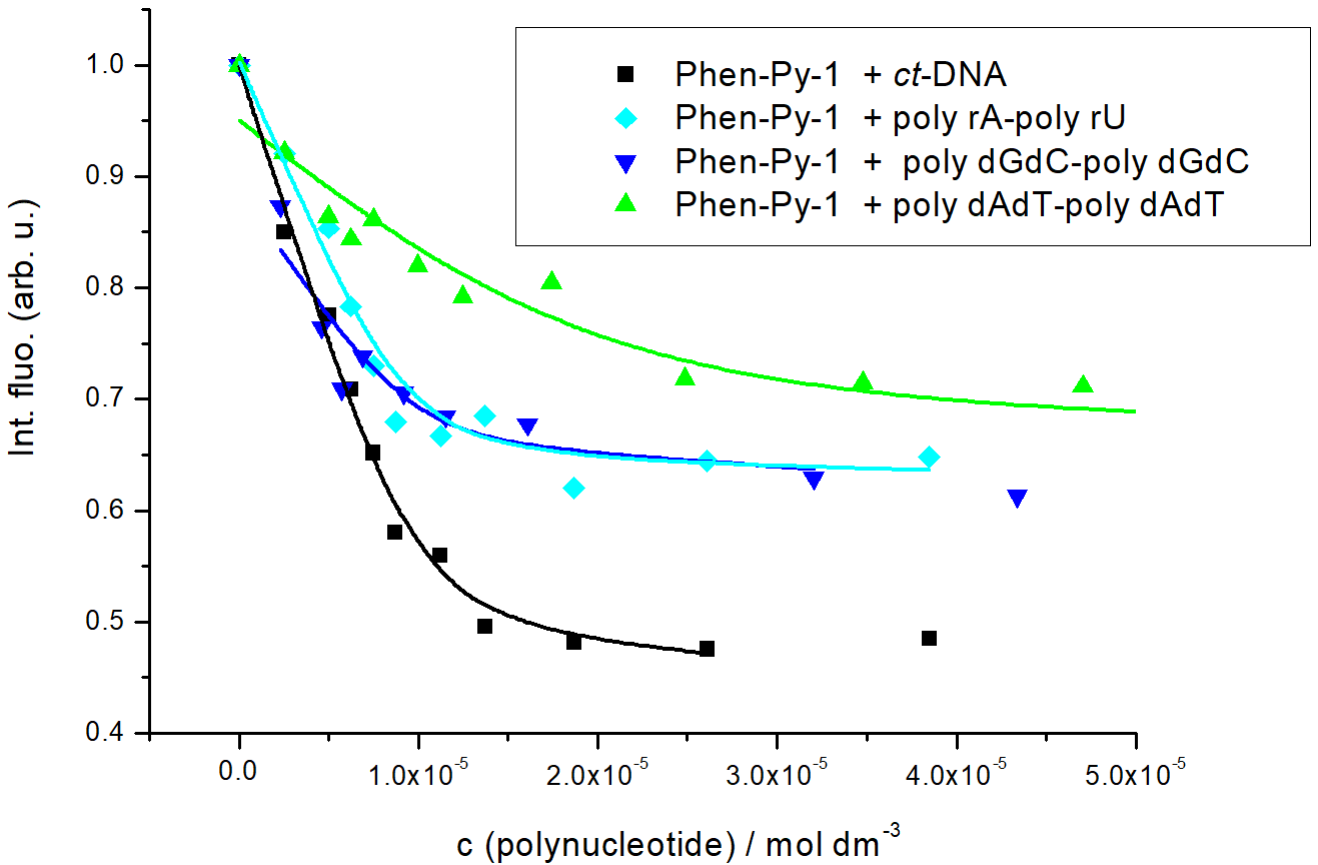
**.** Experimental (■) and calculated (–) (by Scatchard equation Table 2) fluorescence intensities of compound **Phen-Py-1** upon addition of different ds-polynucleotides; fluorescence intensities were normalized for easier comparison. Na cacodylate buffer, pH 7.0, *I_c_* = 0.05 mol dm^−3^, λ_exc_ = 352 nm, λ_em_ = 471 nm.

The excimer fluorescence signal of **Phen-Py-1** (longer linker between phenanthridine and pyrene unit) at 470 nm was quenched up to 30% upon the addition of polynucleotides combined with the significant hypsochromic (blue) shift of the emission maxima (10–20 nm). A similar fluorimetric response concerning the excimer band was obtained independently on polynucleotide’s structure and/or base composition. Fluorescence quenching upon binding was similar to the previously published bispyrene–guanidiniocarbonyl compound, which was found to bind along the phosphate backbone of most of examined DNA/RNA polynucleotides [37]. The monomer fluorescence emission at 400 nm was not changed except for titration with poly dGdC–poly dGdC where a small emission increase of emission at 400 nm was observed. At ratios of an excess of polynucleotide over compound (*r*_[compound]/[polynucleotide]_ < 0.3), spectral changes could be attributed to a single dominant binding mode. Titration data were processed by the Scatchard equation [38–39] and Global Fit procedure [40] to calculate the association constants and ratio *n *_[bound compound]/[polynucleotide]_ (Table 2, Figure 4 and Figures S15–S18, Supporting Information File 1). Compound **Phen-Py-1** showed high, micromolar/submicromolar affinities for all examined polynucleotides.

**Table 2:** Association constants (log *K*_a_)^a^ of complexes of **Phen-Py-1** and **Phen-Py-2** with ds-polynucleotides calculated according to fluorimetric titrations (Na cacodylate buffer, *I_c_* = 0.05 mol dm^−3^, pH 7.0; λ_exc_ = 352 nm; λ_em_ = 370–600 nm, *c* (**Phen-Py-1** and **Phen-Py-2**) = 1–2 × 10^−6^ mol dm^−3^).

	log *K*_a_	
polynucleotide	**Phen-Py-1**	**Phen-Py-2**

ct-DNA	6.97	<4^b^
poly dAdT–poly dAdT	5.97	^c^
poly dGdC–poly dGdC	6.87	^c^
poly rA–poly rU	7.07	^b^

^a^Processing of titration data using Scatchard equation [38–39] and Global Fit procedure [40] gave association constants and values of the ratio *n* = [bound compounds]/[polynucleotide]; for more accessible comparison values of log *K*_a_ for polynucleotide complexes were recalculated for fixed *n* = 0.2; correlation coefficients were >0.9 for all calculated *K*_a_; ^b^small/linear fluorescence change/no fluorescence change the disabled calculation of stability constant; ^c^not determined.

The binding of **Phen-Py-1** to polynucleotides possibly led to the unstacking of intramolecular pyrene–phenanthridine dimer and consequent excimer fluorescence quenching independently of the polynucleotide secondary structure. Concurrently, no monomer fluorescence signal at 400 nm changed, except a small increase upon poly dGdC–poly dGdC addition. Negligible thermal stabilization (Table S1, Figures S6–S8, Supporting Information File 1) did not support the classical intercalation of phenanthridine and/or pyrene moiety. There was the eventual possibility of partial intercalation of the pyrene or phenanthridine unit, but the binding contribution of the phenanthridine or pyrene moiety cannot be discriminated. According to the presented results the most possible binding mode was unspecific electrostatic binding of the dye along the polynucleotide backbone. The reorganized intramolecular conformation of the ligand could explain the quenching of excimer fluorescence and/or redistribution of chromophore charges upon binding since fluorescence quenching is sensitive to factors that affect the rate and probability of contact, including steric shielding and charge–charge interactions [41].

### Circular dichroism (CD) experiments

**Figure 5:**
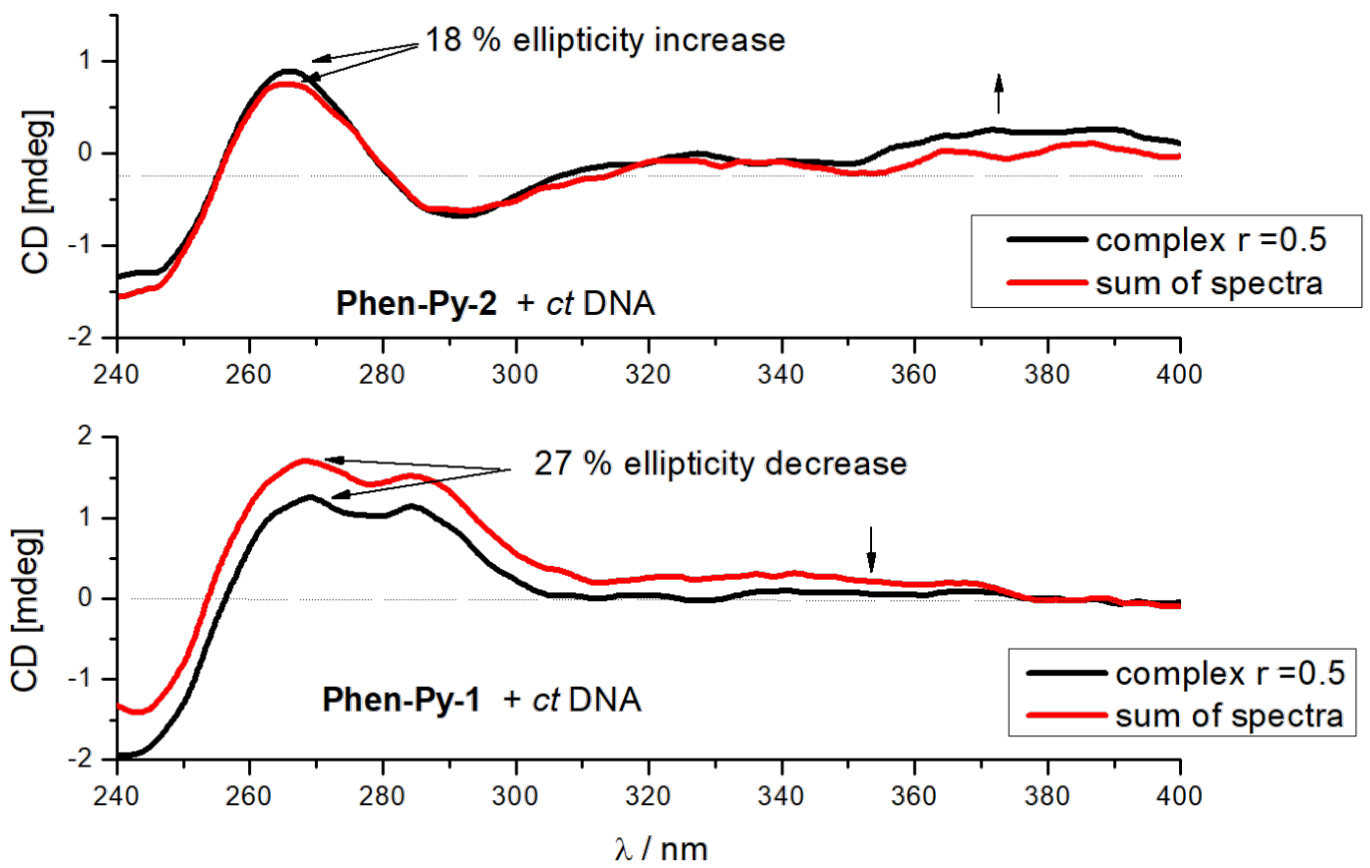
Comparison of spectra of DNA-dye complex (*r* = 0.5, black) and sum of DNA and dye spectra (red) of appropriate concentrations (*c* (*ct*-DNA) = 2 × 10^−5^ mol dm^−3^, *c* (dye) = 1 × 10^−5^ mol dm^−3^, Na cacodylate buffer, pH 7.0, *I_c_* = 0.05 mol dm^−3^).

CD spectroscopy was chosen to monitor conformational changes of polynucleotide's secondary structure induced by small molecule binding [42]. Compounds **Phen-Py-1** and **Phen-Py-2** were built using chiral amino acid building blocks and consequently have an intrinsic CD spectrum. While changes of poly rA–poly rU spectra upon titration with **Phen-Py-1** and **Phen-Py-2** were negligible (Figures S20 and S21, Supporting Information File 1), the addition of **Phen-Py-1** and **Phen-Py-2** to the *ct*-DNA caused the change of the signal at 270 nm (Figure S19, Supporting Information File 1) and at wavelengths longer than 300 nm. Intrinsic spectra of the compound also had to be taken into account. Hence the sum of ct-DNA and **Phen-Py-1** or **Phen-Py-2** CD spectra was compared to CD spectra of the DNA–dye complex at the same concentration (Figure 5).

The complex of **Phen-Py-1**–*ct*-DNA showed a decrease of the CD signal compared to the sum of **Phen-Py-1** and *ct*-DNA spectra, while the complex of **Phen-Py-2**–*ct*-DNA showed a small increase of the CD spectra compared to the sum of **Phen-Py-2** and *ct*-DNA. It was important to note that besides the existence of dyes intrinsic spectra, the UV absorption area of examined dyes (both phenanthridine and pyrene moiety) partly overlapped with the area of DNA/RNA absorption. Therefore, it was difficult to distinguish if the small changes in the 240–290 nm region were caused by a distortion of polynucleotide helicity upon addition of **Phen-Py-1**, or this change was a result of uniform orientation of the dye with respect to DNA chiral axis.

### Binding of **Phen-Py-1** to enzyme dipeptidyl peptidase III in an aqueous medium

**Figure 6:**
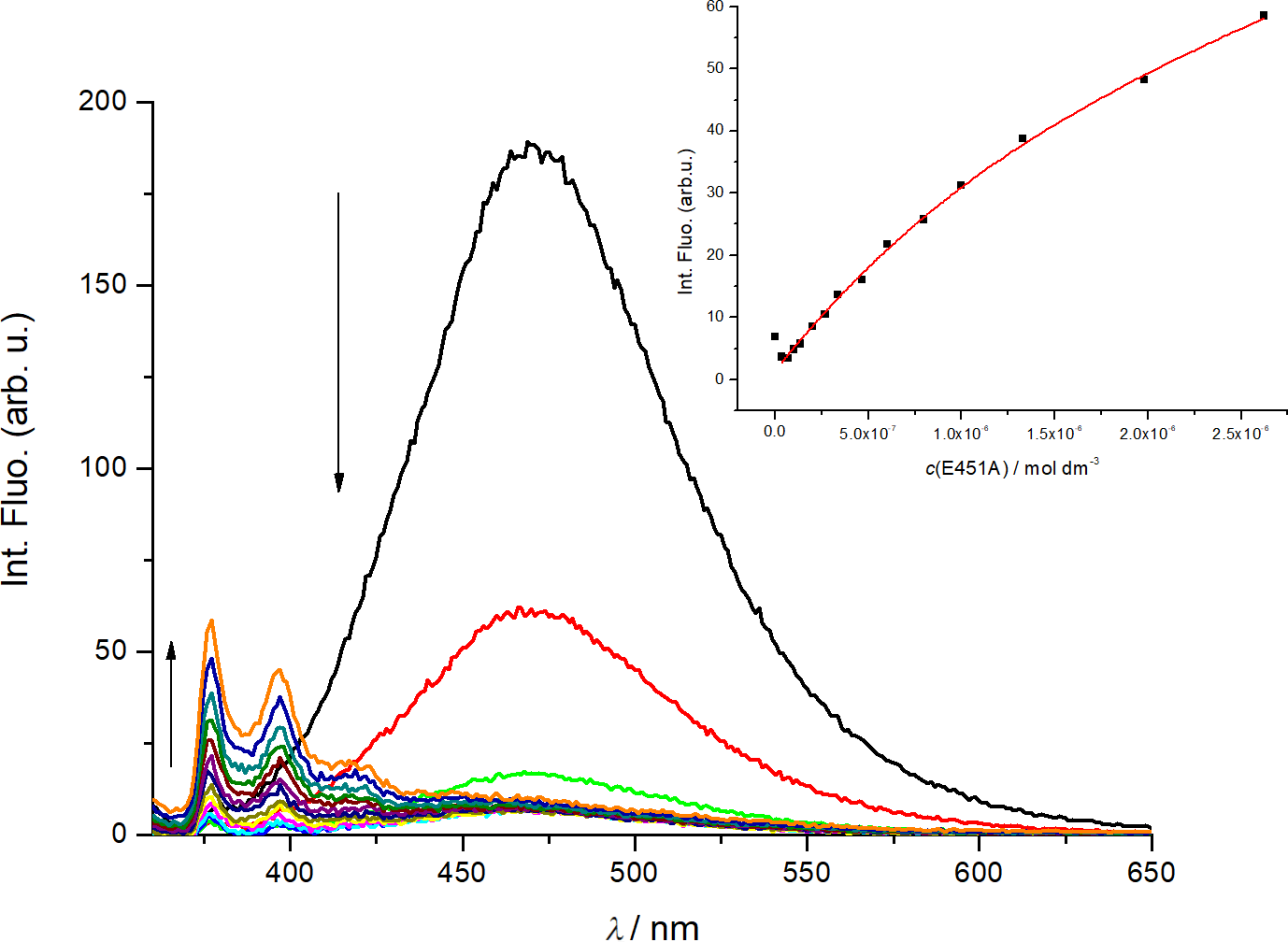
Fluorimetric titration of **Phen-Py-1**, λ_exc_ = 352 nm, *c* = 1 × 10^−6^ mol dm^−3^ with dipeptidyl peptidase (DPPIII) enzyme mutant E451A, inset: Experimental (●) and calculated (—) fluorescence intensities of **Phen-Py-1** at λ_em_ = 377 nm upon addition of dipeptidyl peptidase (DPPIII) enzyme mutant E451A (pH 7.4, Tris-HCl buffer, *I_c_* = 0.02 mol dm^−3^).

Binding of **Phen-Py-1** to dipeptidyl peptidase (DPPIII) inactive enzyme mutant E451A was examined by fluorimetric titrations and ITC titrations and it was found that **Phen-Py-1** binds to the protein with a high affinity (Table 3). This protein is a mono-zinc metalloexopeptidase and hydrolyses dipeptides from the N-termini of substrates that consist of at least three amino acids. DPPIII participates in intracellular protein catabolism, which functions in pain modulation and oxidative stress. These biological functions make DPPIII a valuable target for drug development. Interestingly, although excimer emission was quenched, the pyrene monomer's emission was increased upon addition of the enzyme (Figure 6), suggesting the important role of the hydrophobic pyrene subunit for protein binding [18].

**Figure 7:**
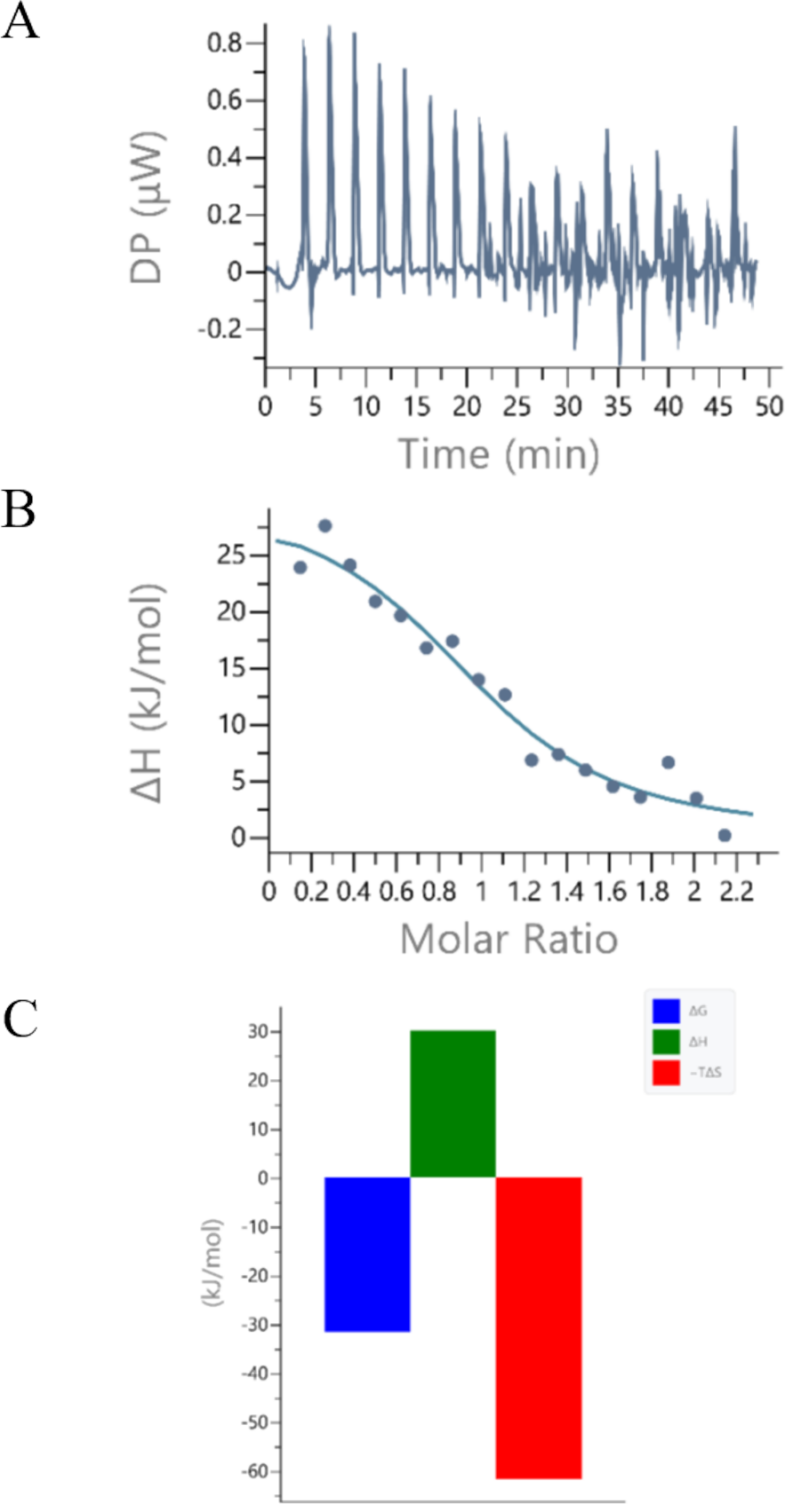
A: ITC titration: raw titration data from the experimental injections of human DPP III enzyme mutant E451A into the solution of **Phen-Py-1**; (pH 7.4, Tris-HCl buffer, *I_c_* = 0.02 mol dm^−3^); B: ITC titration of **Phen-Py-1** with human DPPIII enzyme mutant E451A; experimental data (●) and calculated fit for model one set of sites (–). C: Signature plot for ITC titration of **Phen-Py-1** with human DPP III enzyme mutant E451A.

Association constant and thermodynamical binding parameters were additionally determined by ITC titrations [17]. The resulting titration data were fitted to a single-site binding model (Figure 7, Table 3).

**Table 3:** Association constants (log *K_a_*) of complex of **Phen-Py-1** with human DPP III enzyme mutant E451A calculated according to fluorimetric titrations^a^ and association constants (log *K_a_*) and thermodynamical parameters calculated according to ITC titrations^b^.

	fluorimetric titration^a^	ITC titration^b^			

protein	log *K_a_*	log *K_a_*	Δ_r_*H*/kJ mol^−1^	Δ_r_*G*/kJ mol^−1^	*−T*Δ_r_*S*/kJ mol^−1^
E451A	7.54	5.47 ± 0.14	38.7 ± 9.6	-31.2 ± 0.8	-69.9 ± 8.9

^a^Processing of titration data using Scatchard equation [38–39] gave stability constants; *n* = [bound compounds]/[protein] = 1; correlation coefficients >0.9; Tris-HCl buffer, *I_c_* = 0.02 mol dm^−3^, pH 7.4; λ_exc_ = 352 nm; λ_em_= 370–600, *c*(**Phen-Py-1**) = 1 × 10^−6^ mol dm^−3^. ^b^ITC titration: one aliquot of 0.4 μL and eighteen aliquots of 2 μL of the enzyme human DPP III mutant E451A (*c* = 1.9 × 10^−4^ mol dm^−3^ – 2.4 × 10^−4^ mol dm^−3^) were injected from rotating syringe (500 rpm) into the isothermal cell, containing 200 μL of **Phen-Py-1** (2 × 10^−5^ mol dm^−3^); Tris-HCl buffer, *I_c_* = 0.02 mol dm^−3^, pH 7.4; ϑ = 25.0 °C, 5% DMSO; data were fitted with the model one set of sites with fixed *N* = 1. The result is the mean of three measurements.

ITC experiments confirmed the binding of the ligand with human DPP III mutant E451A. The experiment resulted in positive peaks as a result of an endothermic reaction, which took place with an increase in entropy (Figure 7C, Table 3). This means that the binding is entropically driven, which is generally related to the release of water molecules from the protein to the bulk water [43–44]. The association constants obtained by different methods slightly differ because the measurements were made in a different concentration range. It also can be assumed that the ligand was stacked prior to binding, and unstacked during binding, so entropic loss relating with ligand flexibility was avoided upon binding. Interestingly, the guanidiniocarbonylpyrrole–phenanthridine conjugate bound to human DPP III mutant E451A with the similar thermodynamical pattern as **Phen-Py-1**: endothermic reaction with an increase of entropy. The analogous guanidiniocarbonylpyrrole–pyrene conjugate showed a negative enthalpy change and an increase of entropy [17].

## Conclusion

Two novel phenanthridine–pyrene conjugates **Phen-Py-1** and **Phen-Py-2** were prepared by the condensation of phenanthridinylalanine with the corresponding pyrene-containing carboxylic acid and were linked together by an amide bond. Conjugate **Phen-Py-1** possessed a trimethylene chain linker that allowed pronounced flexibility for the positioning of aromatic units. Although more rigid, **Phen-Py-2** also enabled intramolecular stacking interaction between phenanthridine and pyrene. UV–vis spectra of **Phen-Py-1** and **Phen-Py-2** showed a hypochromic effect at a single wavelength compared to reference compounds with identical chromophores. This noticeable hypochromicity resulted from intramolecular stacking upon the aromatic interaction between phenanthridine and pyrene, which was more pronounced at weakly acidic pH where the phenanthridine nitrogen was protonated. Experimental data agreed well with molecular dynamics simulations, which confirmed that folded structures are more frequent in monoprotonated derivatives **Phen-Py-1^+^** and **Phen-Py-2^+^**, where π–π stacking contacts are further promoted by the favorable cation–π interactions.

Phenanthridine–pyrene conjugate **Phen-Py-1** showed excimer fluorescence that was red shifted compared to the emission of a single phenanthridine or pyrene chromophore. This excimer fluorescence was significantly solvent and pH-dependent. Namely, excimer fluorescence was mostly quenched in methanol, where the monomer fluorescence was also very low. Further, excimer fluorescence was quenched in acidic conditions and increased upon pH increase. Also, excimer emission was quenched upon heating without increasing back after cooling. That was probably caused by the temperature-induced unstacking and also by the aggregation of compounds.

Compound **Phen-Py-2** was more tended both to intramolecular stacking and aggregation. This compound lacked any significant responses upon eventual binding to ds-polynucleotides, both at acidic and neutral pH, except for a small hypochromic change of the CD-spectra upon binding to *ct*-DNA. Opposite of **Phen-Py-2**, more flexible **Phen-Py-1** with longer linker bound strongly, with micromolar and submicromolar affinity, to all examined ds-polynucleotides. Experimental results don't indicate unambiguously particular binding modes of ligands to polynucleotides. Quenching of excimer fluorescence upon addition of ds-polynucleotides combined with a hypsochromic shift of the emission maxima could be explained both by unstacking of the dye and a partial intercalation of one aromatic unit between base pairs, or by unspecific binding of the stacked dye along the polynucleotide backbone. In addition, CD measurements did not give unambiguous results since the polynucleotide and the dye absorbed UV light in the same wavelength region: the small hypochromic change of the *ct*-DNA spectra could be caused both by a decrease in DNA helicity or by the uniform orientation of the dye concerning the chiral axis. Dye **Phen-Py-1** bound to dipeptidyl peptidase (DPPIII) enzyme mutant E451A with high affinity and showed an interesting spectroscopic response: the excimer emission was totally quenched, but the pyrene signal arose. ITC titration revealed **Phen-Py-1** binding to the enzyme as an endothermal, entropically driven event. The ITC titration result suggested an important role of the pyrene subunit, since pyrene had a large hydrophobic surface that preferred binding to the protein. Further, this result could lead to the development of new probes based on pyrene–phenanthridine chromophores that can switch fluorescence signals on/off upon binding to biomacromolecules.

## Experimental

### Synthesis

The phenanthridine derivative of alanine amino acid (**Phen-AA**) has been prepared according to the procedure described earlier [13].

#### General procedure for the synthesis of the compounds **Phen-Py-1** and **Phen-Py-2**

To the solution of Boc-protected amino acid **Phen-AA** [19] in dichloromethane (4 mL) was added a TFA/H_2_O mixture (9:1, v/v; 2 mL) and the reaction was stirred at room temperature for 20 hours. The trifluoroacetate salt of the deprotected amino acid was obtained as yellow oil after evaporation of the solvent. The deprotected compound was then dissolved in anhydrous acetonitrile (3 mL) and appropriate pyrenecarboxylic acid, HBTU, HOBt and Et_3_N were added. The reaction was stirred at room temperature for 20 hours. The products **Phen-Py-1** and **Phen-Py-2** were isolated by preparative thin-layer chromatography in dichloromethane/methanol 9:1.

**Phen-Py-1**: **Phen-AA** (12.3 mg, 0.03 mmol), 1-pyrenebutyric acid (11.2 mg, 0.04 mmol), HBTU (11.4 mg, 0.03 mmol, 98%), HOBt (4.2 mg, 0.03 mmol, 97%) and Et_3_N (16.8 µL, 0.12 mmol) were used according to the general procedure. **Phen-Py-1** was obtained as a white solid (9.4 mg, 56%). mp = 131–132 °C; *R*_f_ = 0,8 (CH_2_Cl_2_/MeOH 9:1); IR (KBr) ν_max_/cm^−1^: 3418 (s), 3294 (s), 3038 (m), 2947 (m), 2858 (m), 1738 (s), 1643 (s), 1582 (m), 1535 (m), 1435 (m), 1377 (m), 1209 (m), 843 (s), 760 (s), 723 (m); ^1^H NMR (CDCl_3_) δ 8.42 (d, *J* = 8.5 Hz, 1H, Phen-10), 8.31 (d, *J* = 8.1 Hz, 1H, Phen-1), 8.21–8.10 (m, 3H, Py), 8.07–7.93 (m, 6H, Phen, 5Py), 7.88 (d, *J* = 1.5 Hz, 1H, Phen-4), 7.73 (d, *J* = 7.8 Hz, 1H, Py), 7.66–7.45 (m, 3H, Phen), 5.98 (d, *J* = 7.7 Hz, 1H, NH), 5.12–5.03 (dd, *J* = 13.7, 6.0 Hz, 1H, CH), 3.75 (s, 3H, OCH_3_), 3.48–3.39 (m, 1H, CH_2_), 3.36–3.18 (m, 3H, CH_2_) 2.92 (s, 3H, Phen-CH_3_), 2.38–2.24 (m, 2H, CH_2_), 2.22–2.09 (m, 2H, CH_2_) ppm; ^13^C NMR (CDCl_3_) δ 172.4 (C_q_), 172.1 (C_q_), 158.4 (C_q_), 143.7 (C_q_), 135.6 (C_q_), 135.3 (C_q_), 131.8 (CH-Ar), 131. 7 (C_q_), 131.5 (C_q_), 131.0 (C_q_), 130.1 (C_q_), 129.4 (CH-Ar), 128.8 (C_q_), 128.7 (CH-Ar), 127.6 (CH-Ar), 127.5 (CH-Ar), 127.3 (CH-Ar), 126.9 (CH-Ar), 126.8 (CH-Ar), 126.5 (CH-Ar), 126.0 (CH-Ar), 125.0 (CH-Ar), 124.9 (CH-Ar), 124.9 (CH-Ar), 123.6 (C_q_), 123.3 (CH-Ar), 122.9 (CH-Ar), 121.9 (CH-Ar), 53.2 (CH-Ala), 52.7 (OCH_3_), 38.4 (CH_2_), 36.1 (CH_2_), 32.8 (CH_2_), 27.3 (CH_2_), 23.5 (CH_3_) ppm; HRMS (*m/z)*: [M + H]^+^ calcd. for C_38_H_32_N_2_O_3_^+^, 565.2485; found, 565.2464.

**Phen-Py-2**: **Phen-AA** (12.0 mg, 0.03 mmol), 1-pyrenecarboxylic acid (9.2 mg, 0.04 mmol), HBTU (11.6 mg, 0.03 mmol, 98%), HOBt (4.2 mg, 0.03 mmol, 97%) and Et_3_N (16.8 µL, 0.12 mmol) were used according to the general procedure. **Phen-Py-2** was obtained as a white solid (15.9 mg, 84%). mp = 230–231 °C; *R*_f_ = 0.8 (CH_2_Cl_2_:MeOH 9:1); IR (KBr) ν_max_/cm^−1^: 3435 (s), 3261 (s), 1740 (m), 1634 (s), 1531 (m), 849 (m), 760 (m); ^1^H NMR (CDCl_3_) δ 8.59 (d, *J* = 8.5 Hz, 1H, Phen-10), 8.51 (d, *J* = 7.3 Hz, 1H, Phen-1), 8.36 (d, *J* = 9.3 Hz, 1H, Py), 8.21 (d, *J* = 7.4 Hz, 1H, Py), 8.18–7.98 (m, 8H, 2Phen, 6Py), 7.91 (d, *J* = 9.3 Hz, 1H, Py), 7.78–7.67 (m, 2H, Phen), 7.66–7.58 (m, 1H, Phen), 6.66 (d, *J* = 7.6 Hz, 1H, NH), 5.51–5.40 (m, 1H, CH-Ala), 3.89 (s, 3H, OCH_3_), 3.81–3.72 (m, 1H, CH_2_-Ala), 3.57–3.47 (m, 1H, CH_2_-Ala), 2.90 (s, 3H, Phen-CH_3_) ppm; ^13^C NMR (CDCl_3_) δ 172.1 (C_q_), 169.5 (C_q_), 158.7 (C_q_), 135.6 (C_q_), 133.1 (C_q_), 132.1 (CH-Ar), 131.3 (C_q_), 130.7 (C_q_), 129.9 (CH-Ar), 129.4 (CH-Ar), 129.1 (CH-Ar), 129.1 (CH-Ar), 128.9 (CH-Ar), 127.4 (CH-Ar), 127.2 (CH-Ar), 126.7 (CH-Ar), 126.6 (CH-Ar), 126.1 (CH-Ar), 126.1 (CH-Ar), 124.6 (CH-Ar), 124.5 (CH-Ar), 124.2 (CH-Ar), 123.7 (C_q_), 123.1 (CH-Ar), 122.1 (CH-Ar), 54.0 (CH-Ala), 52.9 (OCH_3_), 38.5 (CH_2_), 23.4 (CH_3_) ppm; HRMS (*m/z)*: [M + H]^+^ calcd. for C_35_H_26_N_2_O_3_^+^, 523.2022; found, 523.2025.

### Study of DNA/RNA and enzyme interactions

**General procedures:** Solvents were distilled from appropriate drying agents shortly before use. TLC was carried out on DC-plastikfolien Kieselgel 60 F_254_ and preparative thick-layer (2 mm) chromatography was done on Merck 60 F_254_. ^1^H and ^13^C NMR spectra were recorded in DMSO-*d*_6_ or CDCl_3_ on Bruker AV 300 and 600 MHz spectrometers using TMS as the internal standard. The assignment of C-atoms and protons were confirmed on the basis of 2D NMR HETCOR, COSY, and NOESY. Chemical shifts (δ) are expressed in ppm, and *J* values in Hz. Signal multiplicities are denoted as s (singlet), d (doublet), t (triplet), q (quartet) and m (multiplet). High-resolution mass spectra (HRMS) were obtained using a MALDI–TOF/TOF mass spectrometer 4800 Plus MALDI TOF/TOF analyzer (Applied Biosystems Inc., Foster City, CA, USA). The electronic absorption spectra of newly prepared compounds, UV–vis titration and thermal melting experiments were measured on a Varian Cary 100 Bio spectrometer. Fluorescence spectra were recorded on a Varian Cary Eclipse fluorimeter. CD spectra were recorded on JASCO J815 spectrophotometer. Absolute quantum yields (Φ_f_) were determined using software implemented with the instrument by the Integrating sphere SC-30 of the Edinburgh FS5 spectrometer. Quantum yields were measured for argon-purged solutions in sodium cacodylate buffer, pH 7.0, *I* = 0.05 mol dm^−3^, or pH 7.0, *I_c_* = 0.05 mol dm^−3^ (λ_exc_= 280 nm) at room temperature (25 °C) in a quartz cuvette of 10 mm path length; to avoid the scattering of incident light at the liquid–air interface, testing solutions with a 2 mL volume were used. Fluorescence and CD spectra were recorded using appropriate 1 cm path quartz cuvettes; UV–vis spectra were recorded in 1 cm path quartz cuvettes or using an immersion probe with 5 cm light path length. Isothermal titration calorimetry (ITC) titrations were performed on a Malvern PEAQ-ITC microcalorimeter (MicroCal, Inc.,Northampton, MA, USA). MicroCal PEAQ-ITC analysis software, supplied by the manufacturer, was used for data analysis. Polynucleotides were purchased as noted: calf thymus (*ct*)-DNA, poly dAdT–poly dAdT, poly dGdC–poly dGdC and poly rA–poly rU (Sigma) and dissolved in sodium cacodylate buffer, *I_c_* = 0.05 mol dm^−3^, pH 7.0. The calf thymus (*ct*) DNA was additionally sonicated and ﬁltered through a 0.45 µm ﬁlter [45]. The polynucleotide concentration was determined spectroscopically and expressed as the concentration of phosphates [45–46]. Recombinant human DPP III was obtained by heterologous expression in *Escherichia coli* and purification according to Špoljarić et al. [47–48]. Stock solutions of **Phen-Py-1** and **Phen-Py-2** were prepared by dissolving the compounds in DMSO; the total DMSO content was below 1% in UV–vis and below 0.1% in fluorimetric measurements. All measurements were performed in sodium cacodylate buffer, *I_c_* = 0.05 mol dm^−3^.

**UV–vis, CD, and fluorescence titrations:** UV–vis and fluorimetric titrations were performed by adding portions of polynucleotide solution into the solution of the studied compound. After mixing polynucleotides/protein with studied compounds it was observed that the equilibrium was reached in less than 120 seconds. Compounds **Phen-Py-1** and **Phen-Py-2** showed a decrease of their excimer fluorescence emission intensity upon time. Therefore, buffer solutions of compounds were prepared 24 hours before titration with polynucleotides to ensure stable spectra of compounds. In fluorimetric titrations, the concentrations of studied **Phen-Py-1** and **Phen-Py-2** were 2 × 10^−6^ mol dm^−3^. An excitation wavelength of λ_exc_ = 352 nm was used for titrations to avoid absorption of excitation light caused by increasing absorbance of the polynucleotide or protein. The emission was measured in the range of λ_em_ = 350–650 nm. Fluorescence spectra were collected at *r* < 0.3 (*r* = [compound]/[polynucleotide]) to assure one dominant binding mode. Titration data were processed by means of Scatchard equation [38] and Global Fit procedure [40]. Calculations mostly gave values of ratio *n* = 0.2 ± 0.05, but for easier comparison all *K*_a_ values were re-calculated for fixed *n* = 0.2. Values for *K*_a_ have satisfactory correlation coefficients (>0.98). In Scatchard equation values of association constant (*K*_a_) and ratio (*n* = [bound compound]/[polynucleotide]) are highly mutually dependent and similar quality of fitting calculated to experimental data is obtained for ±20% variation for *K*_s_ and *n*; this variation can be considered as an estimation of the errors for the given binding constants. CD experiments were performed by adding portions of **Phen-Py-1** and **Phen-Py-2** compound stock solution into the solution of polynucleotide (*c* ≈ 1–2 × 10^−5^ mol dm^–3^). The examined **Phen-Py-1** and **Phen-Py-2** compounds were chiral and therefore possessed intrinsic CD spectra. CD spectra were recorded with a scanning speed of 200 nm/min. Buffer background was subtracted from each spectrum, thus each spectrum was a result of two accumulations.

**Thermal melting experiments:** Thermal melting curves for ds-DNA, ds-RNA and their complexes with studied compounds were determined by following the absorption change at 260 nm as a function of temperature. The absorbance scale was normalized. *T*_m_ values were the midpoints of the transition curves determined from the maximum of the first derivative and checked graphically by the tangent method. The Δ*T*_m_ values were calculated subtracting *T*_m_ of the free nucleic acid from *T*_m_ of the complex. Every Δ*T*_m_ value here reported was the average of at least two measurements. The error in Δ*T*_m_ is ±0.5 °C.

**Isothermal titration calorimetry (ITC) experiments**: A non-covalent interaction study of **Phen-Py-1** with the human enzyme mutant DPP III E451A was performed on a MicroCal PEAQ-ITC microcalorimeter (Malvern, UK). Measurements were made in 20 mM Tris-HCl buffer, pH 7.4 at 25.0 °C with the addition of 5% DMSO. All experiments were performed under the same conditions; temperature 25.0 °C, reference power 30.0 μW, high feedback, stirring speed 500 rpm and initial delay 60 s. The enzyme solution (190–240 μM) was in the syringe (40 μL) and the compound **Phen-Py-1** (20 μM) was in the reaction cell (200 μL). The reaction was started with a 0.4 μL injection of enzyme followed by 18 injections 2.0 μL each, with 150 seconds spacing to allow for equilibration. Blank experiments were carried out to determine the heats of dilution of the ligand and the enzyme. The resulting data were analyzed by using MicroCal PEAQ-ITC analysis software, supplied by the manufacturer, according to the model based on a single set of identical binding sites to estimate the binding constants (*K*_a_) and the enthalpy of binding (∆_r_*H*). The reaction Gibbs energies (∆_r_*G*) were calculated by using the following equation: ∆_r_*G* = −*RT*ln(*K*_a_). The entropic contribution to the binding Gibbs energy was calculated by the equation: *T*∆_r_*S* = ∆_r_*H* − ∆_r_*G*.

**Confocal microscopy:** HeLa cells were cultured and maintained in complete high glucose (4.5 g/L) Dulbecco's Modified Eagle's Medium (DMEM, Sigma Aldrich) with the addition of 10% fetal bovine serum (FBS), 1% non-essential amino acids and 1% antibiotic/antimycotic solution (all chemicals were purchased by Capricorn Scientific GmbH). The cells were kept at 37 °C and 5% CO_2_ in a Heracell 150 humidified incubator (Heraeus, Germany). Before confocal microscopy experiments, HeLa cells were counted on LUNA-II Automated Cell Counter (Logos Biosystems) and transferred to 4-chamber 35 mm glass-bottom dishes (IBL, Austria) at a concentration of 15.000 cells per chamber and grown overnight. The dye **Phen-Py-1** was added to the cells at a final concentration of 1 × 10^−6^ M, an hour before confocal imaging.

**Computational details:** In order to sample the conformational flexibility of investigated systems and probe their intrinsic dynamics in the aqueous solution, classical molecular dynamics (MD) simulations were performed employing standard generalized AMBER force fields (ff14SB [49] and GAFF [50]) as implemented within the AMBER16 program package [51]. All structures were subsequently solvated in a truncated octahedral box of TIP3P water molecules spanning a 10 Å thick buffer of solvent molecules around each system, and submitted to periodic simulations where the excess positive charge was neutralized with an equivalent number of chloride anions in monoprotonated systems corresponding to pH 5. Upon gradual heating from 0 K, MD simulations were performed at 300 K for a period of 300 ns, maintaining the temperature constant using the Langevin thermostat with a collision frequency of 1 ps^−1^. The obtained structures in the corresponding trajectories were clustered based on DBSCAN density-based algorithm according to recommended procedures. The idea behind this computational strategy was to investigate whether intrinsic dynamical features of studied conjugates both affect and can explain their tendency to undergo mutual association and form stacking interactions. The mentioned approach recently turned out as very useful in interpreting the affinities of several nucleobase – guanidiniocarbonyl–pyrrole conjugates towards single stranded RNA systems [19,52].

To confirm that the described clustering analysis elucidated the most representative structures of each conjugate at both experimental pH values, we proceeded by calculating energies of the excited states responsible for the experimental UV–vis spectra corresponding to isolated conjugates in the aqueous solution. For that purpose, we used the most abundant structure of each system in Figure 2 and performed the geometry optimization by the M06-2X DFT approach [53] together with the 6–31+G(d) basis set [54] in the Gaussian 16 program package [55], with the water solvent effects modeled through the implicit SMD solvation [56]. The choice of such computational setup was prompted by its success in reproducing various features of different organic [57–58], organometallic [59], and protein systems [60], being particularly accurate for relative trends among similar systems, which is the focus here. This was followed by the TD-DFT computations at the same level of theory considering 32 lowest singlet electronic excitations. The choice of this setup was prompted by its recent success in modeling UV–vis spectra of organic and inorganic systems in various solvents [61–63].

## Supporting Information

File 1 Additional experimental data.
