# Supplementary material for: Phenanthridine–pyrene conjugates as fluorescent probes for DNA/RNA and an inactive mutant of dipeptidyl peptidase enzyme
Source: Beilstein J Org Chem. 2023 Apr 26;19:550–65. doi: 10.3762/bjoc.19.40 (PMC10155618; doi:10.3762/bjoc.19.40)
Supplement: File 1 — Additional experimental data. [file Beilstein_J_Org_Chem-19-550-s001.pdf]

BEILSTEIN JOURNAL OF ORGANIC CHEMISTRY

Supporting Information

for

Phenanthridine–pyrene conjugates as fluorescent probes for DNA/RNA and an inactive mutant of dipeptidyl peptidase enzyme

Josipa Matić, Tana Tandarić, Marijana Radić Stojković, Filip Šupljika, Zrinka Karačić, Ana Tomašić Paić, Lucija Horvat, Robert Vianello and Lidija-Marija Tumir

Beilstein J. Org. Chem. **2023,** *19,* 550–565. doi:10.3762/bjoc.19.40

Additional experimental data

License and Terms: This is a supporting information file under the terms of the Creative Commons Attribution License (https://creativecommons.org/licenses/by/4.0). Please note that the reuse, redistribution and reproduction in particular requires that the author(s) and source are credited and that individual graphics may be subject to special legal provisions.
The license is subject to the *Beilstein Journal of Organic Chemistry* terms and conditions: (https://www.beilstein-journals.org/bjoc/terms)

Content

1. Spectroscopic properties of **Phen-Py-1** and **Phen-Py-2**

2. Interactions of **Phen-Py-1** and **Phen-Py-2** with DNA/RNA and protein

3. NMR spectra of **Phen-Py-1** and **Phen-Py-2**

4. Computational analysis

5. Confocal microscopy

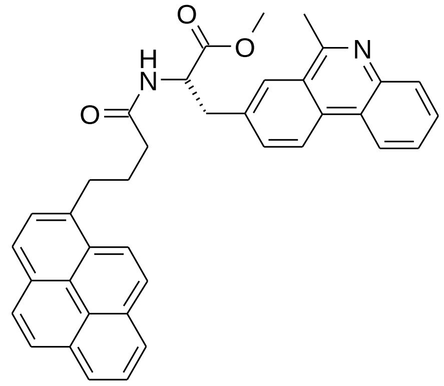 **Phen-Py-1**	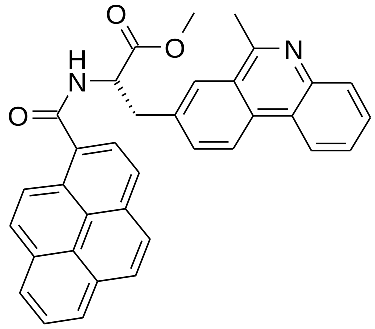 **Phen-Py-2**

Scheme 1. Structures of examined **Phen-Py-1** and **-2**.

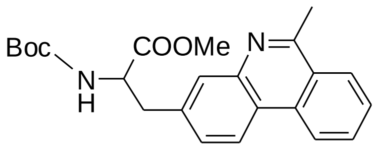 **Phen-AA**	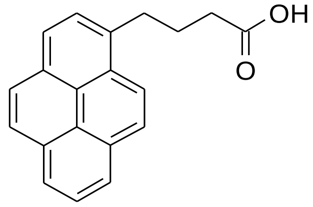 **1-Pyrenebutyric acid (PBA)**

Scheme 2. Structures of reference compounds.

1. Spectroscopic properties of Phen-Py-1 and -2

Studied compounds were moderately soluble in DMSO (up to $c = 1 \times 10^{-3}$ mol dm^{-3}). DMSO stock solutions of compounds were stable during few months. All measurements were recorded in Na-cacodylate buffer (I_c = 0.05mol dm^{-3}) both at pH 5.0 and pH 7.0. Volume ratio of DMSO was less than 1% in all measurements.

UV–vis spectra of examined compounds were recorded in Na-cacodylate buffer (I_c = 0.05mol dm^{-3}) both at pH 5 and pH 7. Further, spectra were recorded using immersion probe with 5 cm light path length, which allowed measurements at concentration range $5 \times 10^{-7} - 3 \times 10^{-6}$ mol dm^{-3} to avoid self-aggregation. Absorbancies of aqueous solutions of compounds **Phen-Py-1** and **2** were proportional to their concentrations up to concentrations $c = 3 \times 10^{-6}$ mol dm^{-3}. Thermal dependent UV–vis spectra

were recorded using quartz cuvettes (1 cm path). UV–vis spectra of aqueous solutions of examined compounds **1**–**2** showed minor irreversible changes upon heating that were attributed to precipitation. Absorption maxima and corresponding molar extinction coefficients (ε) were given in Table 1 and Figure S1.

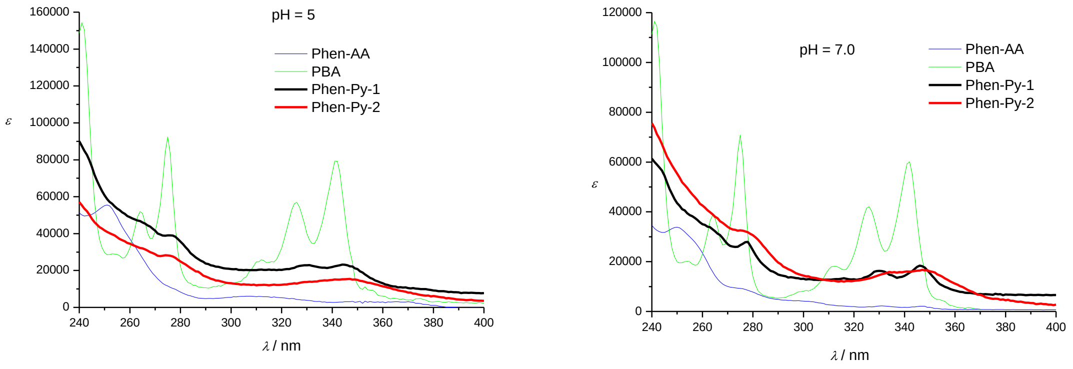

Figure S1. UV–vis spectra of **Phen-Py-1** and **2** and reference compounds **Phen-AA** and **PBA** at pH 5 and pH 7

Fluorescence emission of **Phen-Py-1** and **-2** measured at pH 5 and pH 7 (cacodylate buffer, I_c =0.05 mol dm^{-3}) was linearly dependent on the concentration up to 3 $\times$ 10^{-6} mol dm^{-3} (Figure S2).
Fluorescence intensity was decreased upon time and also upon temperature increase without reproducibility after cooling back. That was attributed to the temperature-induced unstacking of phenanthridine and pyrene but also aggregation/precipitation of the compounds.

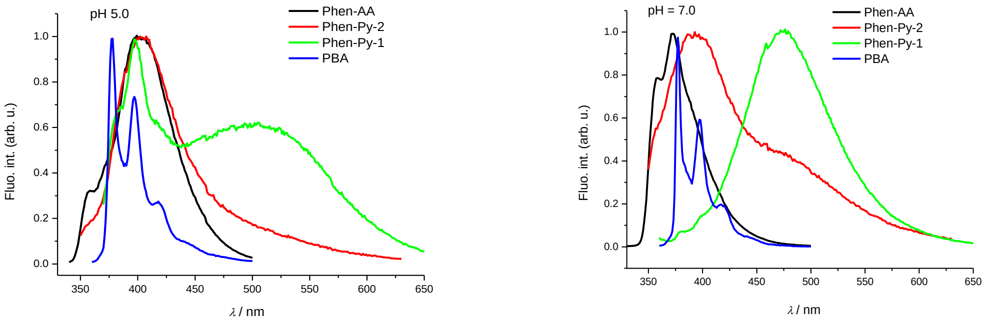

Figure S2. Normalized fluorescence emission spectra of **Phen-Py-1** (λ_{exc} = 350 nm), **Phen-Py-2** (λ_{exc} = 350 nm), reference Phen-AA (λ_{exc} = 250 nm) and reference PBA (λ_{exc} = 342 nm) compounds (sodium cacodylate buffer, I_c = 0.05 mol dm^{-3}, left: pH 5 right: pH 7)

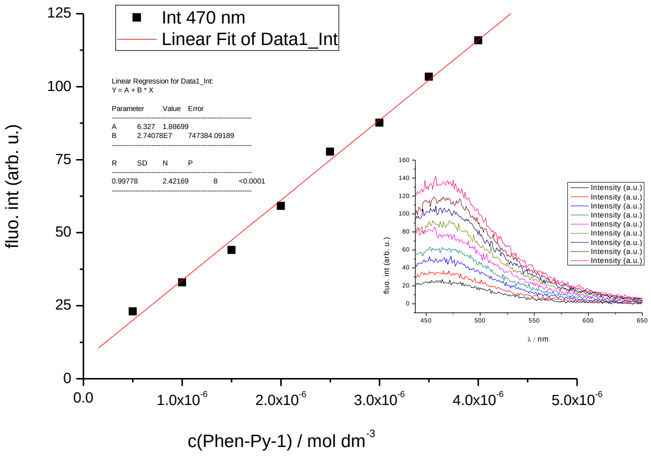

Figure S3 Linear dependence (—) of the fluorescence emission intensity of **Phen-Py-1** at 470 nm (■) Inset: Excimer emission spectra of **Phen-Py-1** (λ_{exc} = 350 nm, sodium cacodylate buffer, I_c = 0.05 mol dm^{-3}, pH 7)

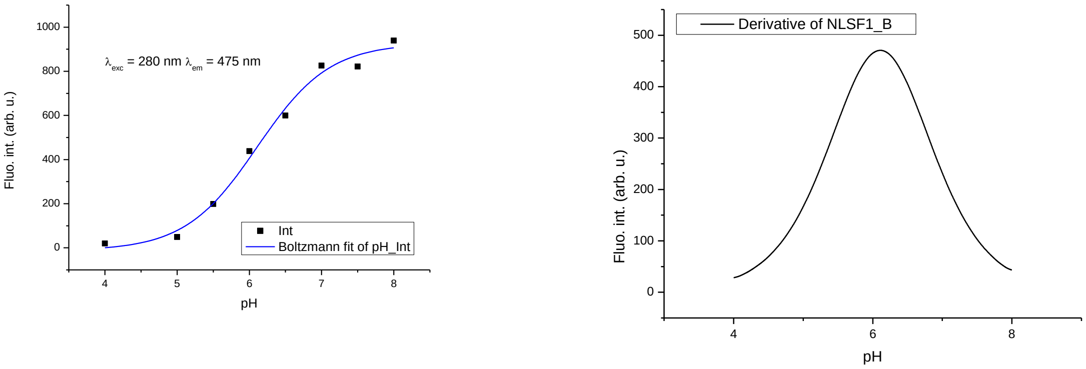

Figure S4. Left: Fluorescence emission spectra (λ_{exc} = 280 nm; λ_{em} = 475 nm)of **Phen-Py-1** (c = 2 × 10^{-6} mol dm^{-3}) at different pH values (Na-cacodylate, HCl, I_c = 0.05 mol dm^{-3} at 25 °C); Right: First derivation of fluorescence emmision at 475 nm in dependence of pH.

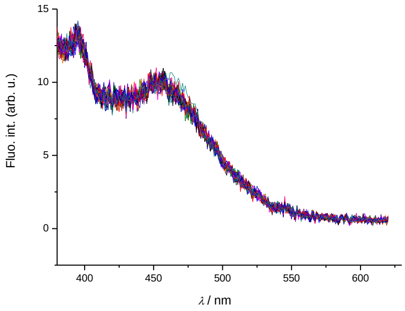

Figure S5. Fluorescence emission spectra of **Phen-Py-1** (c = 2 × 10^{-6} mol dm^{-3}) measured during 60 cycles every 2 minutes at 25 °C, λ_{exc} = 352 nm in methanol.

2. Study of interactions of Phen-Py-1 and -2 with ds-DNA and ds-RNA in aqueous media

2.1. Thermal melting experiments

Non-covalent binding of small molecules to ds-polynucleotides usually has a certain effect on the thermal stability of helices thus giving different T_m values (temperature of dissociation of double stranded helix into two single stranded polynucleotides)[1]. Difference between T_m value of free polynucleotide and complex with a small molecule (ΔT_m value) is an important factor in the characterisation of small molecule / *ds*-polynucleotide interactions. All thermal melting experiments were performed sodium cacodylate buffer, pH 7.0, $I_c = 0.05$ mol dm^{-3}.

At pH 7 **Phen-Py-1** and **Phen-Py-2** didn't thermally stabilize any *ds*-DNA/RNA (Table S1,Figure S6-S8).

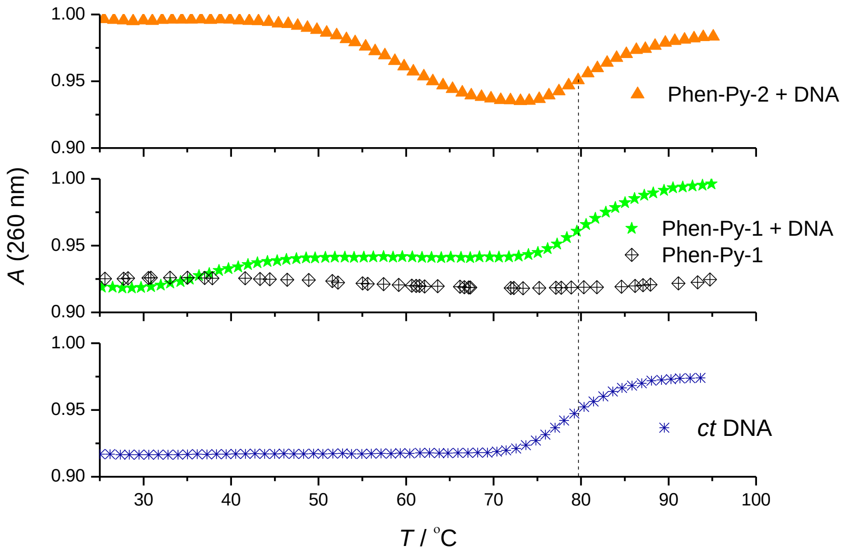

Figure S6. Melting curves of **Phen-Py-1** ($c = 5 \times 10^{-6}$ mol dm^{-3}) and *ct*-DNA upon addition of **Phen-Py-1** and **Phen-Py-2** (c(DNA)= 2×10^{-5} mol dm^{-3}; ratio r[compound] / [polynucleotide] = 0.3) at pH 7.0 (sodium cacodylate buffer, $I_c = 0.05$ mol dm^{-3}).

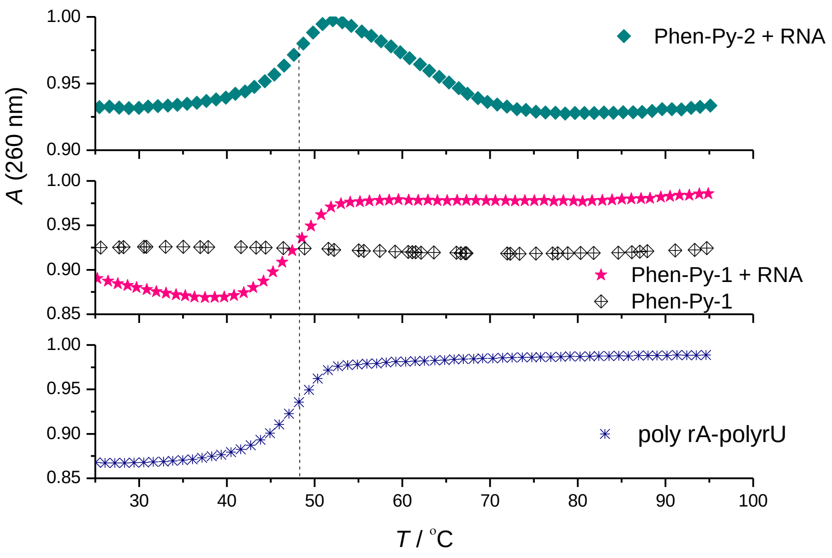

Figure S7. Melting curves of **Phen-Py-1** (c= 5 × 10^{-6} mol dm^{-3}) and poly rA-poly rU upon addition of **Phen-Py-1** and **Phen-Py-2** (c(RNA)= 2 × 10^{-5} mol dm^{-3}; ratio r[compound] / [polynucleotide] = 0.3) at pH 7.0 (sodium cacodylate buffer, I_c = 0.05 mol dm^{-3}).

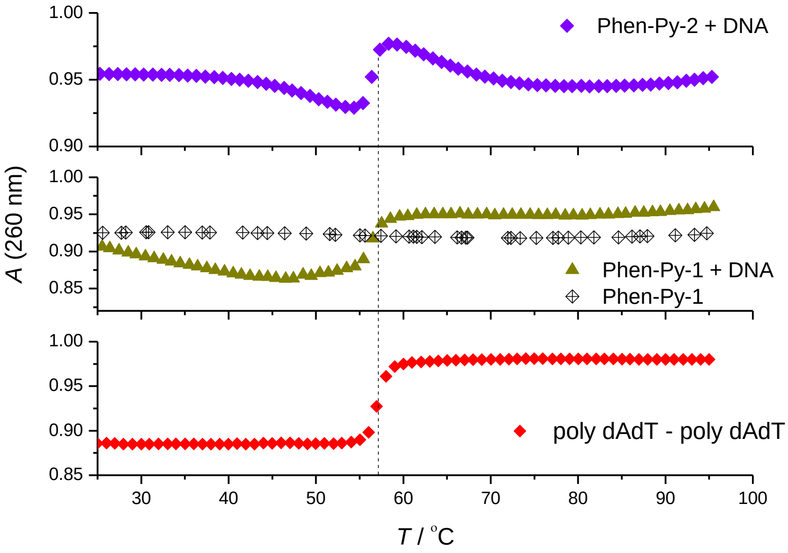

Figure S8. Melting curves of **Phen-Py-1** (c= 5 × 10^{-6} mol dm^{-3}) and poly dAdT – poly dAdT upon addition of **Phen-Py-1** and **Phen-Py-2** (c (DNA) = 2 × 10^{-5} mol dm^{-3}; ratio r[compound] / [polynucleotide] = 0.3) at pH 7.0 (sodium cacodylate buffer, I_c = 0.05 mol dm^{-3}).

Table S1. The [a]ΔT_m values (°C) of studied ds-polynucleotides upon addition of **Phen-Py-1** and **Phen-Py-2** (ratio r^b = 0.3) at pH 7.0 (buffer sodium cacodylate, I_c = 0.05 mol dm^{-3}), c(DNA / RNA) = 1–2 × 10^{-5} mol dm^{-3}.

	**ΔT_m / °C**		
**Compound**	***ct*-DNA**	**poly dAdT – poly dAdT**	**poly rA – poly rU**
**Phen-Py-1**	0	0	0
**Phen-Py-2**	precipitation	0	precipitation

[a] Error in ΔT_m : ± 0.5°C;

[b] *r* = [compound] / [polynucleotide];

[d] not determined due to precipitation

2.1. Spectrophotometric titrations

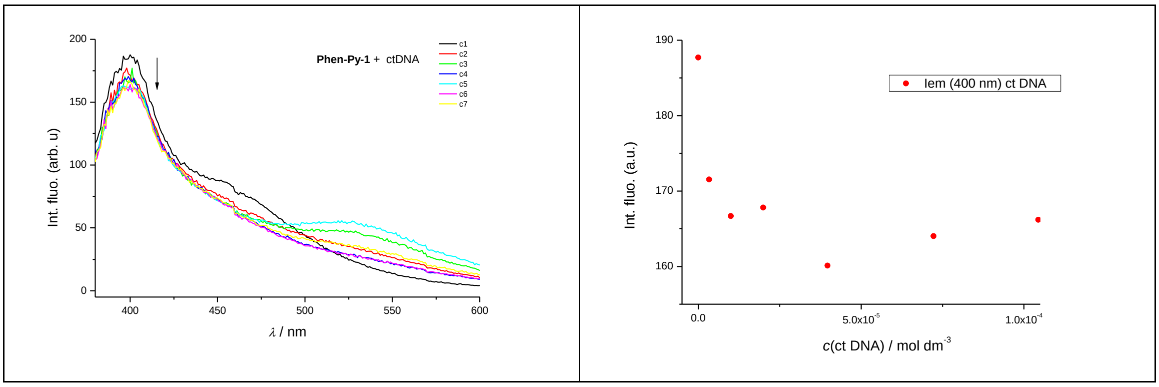

Figure S9. Left: Fluorimetric titration of **Phen-Py-1** with *ct*-DNA, λ_{exc} = 352 nm, c = 2 × 10^{-6} mol dm^{-3}, Right: Experimental (●) fluorescence intensities of **Phen-Py-1** at λ_{em} = 400 nm upon addition of *ct*-DNA (**pH 5.0**, Na cacodylate buffer, I_c = 0.05 mol dm^{-3}).

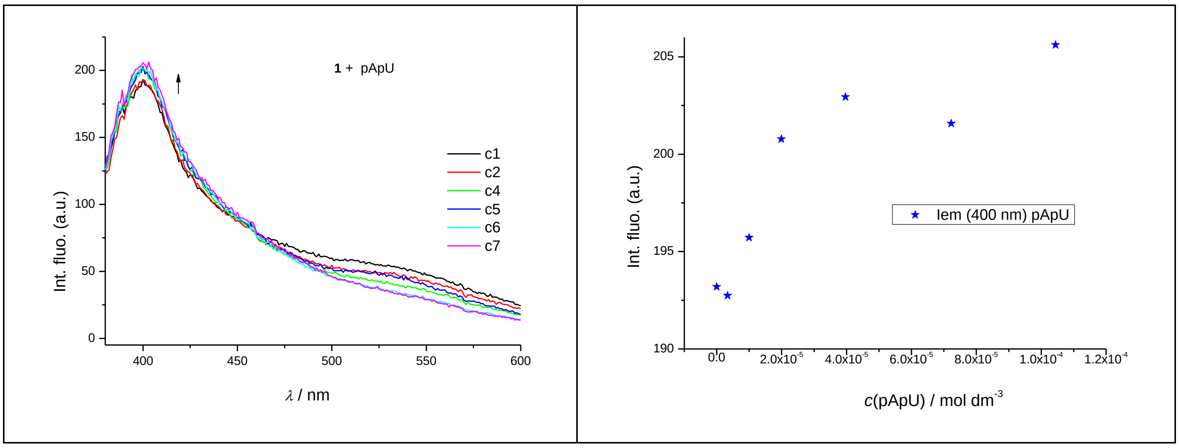

Figure S10. Left: Fluorimetric titration of **Phen-Py-1**, λ_{exc} = 352 nm, c = 2 × 10^{-6} mol dm^{-3} with poly rA-poly rU, Right: Experimental (●) fluorescence intensities of **Phen-Py-1** at λ_{em} = 400 nm upon addition of poly rA-poly rU (**pH 5.0**, Na cacodylate buffer, I_c = 0.05 mol dm^{-3}).

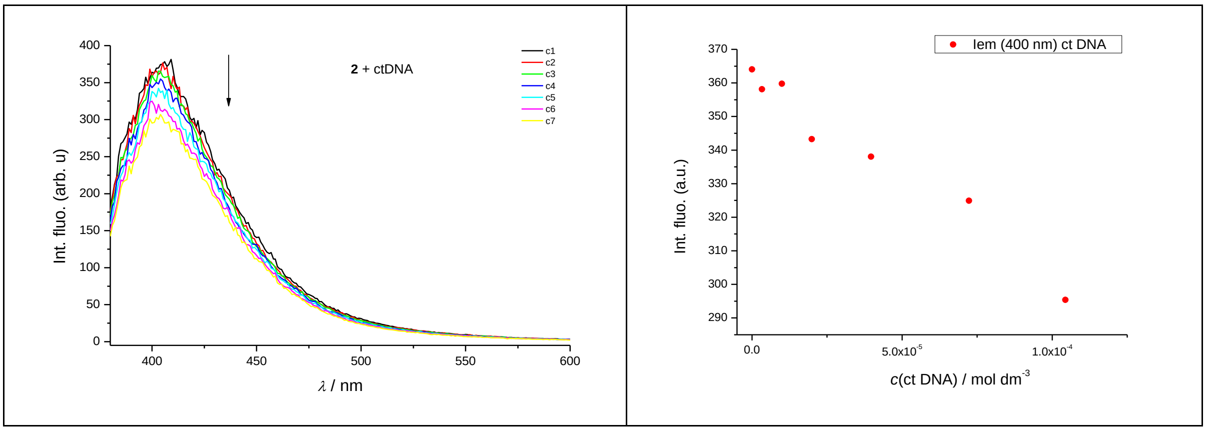

Figure S11. Left: Fluorimetric titration of **Phen-Py-2**, λ_{exc} = 352 nm, $c = 2 \times 10^{-6}$ mol dm^{-3} with *ct*-DNA, Right: Experimental (●) fluorescence intensities of **Phen-Py-2** at λ_{em} = 400 nm upon addition of *ct*-DNA (**pH 5.0**, Na cacodylate buffer, I_c = 0.05 mol dm^{-3}).

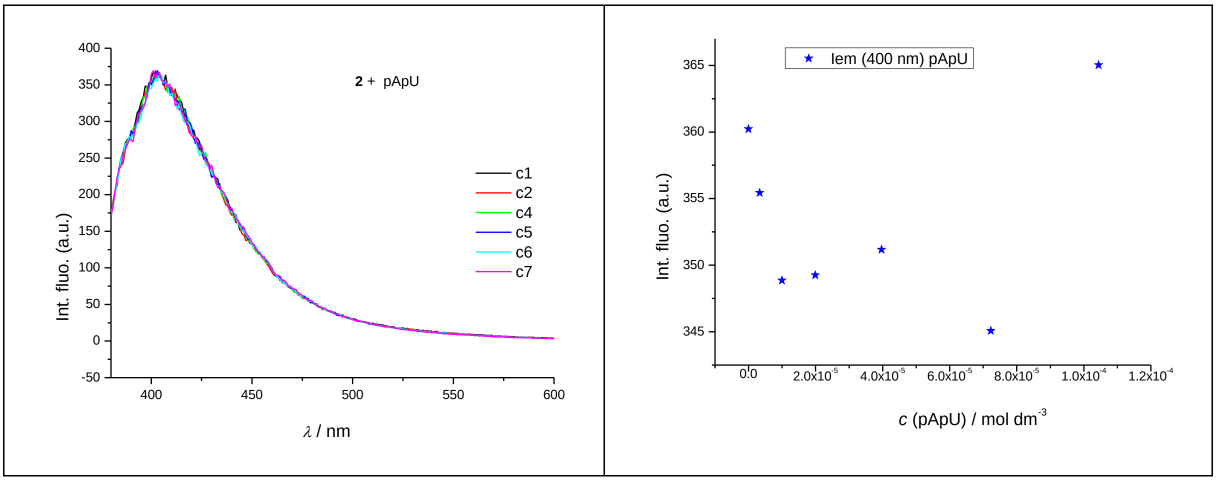

Figure S12. Left: Fluorimetric titration of **Phen-Py-2**, λ_{exc} = 352 nm, $c = 2 \times 10^{-6}$ mol dm^{-3} with poly rA-poly rU, Right: Experimental (●) fluorescence intensities of **Phen-Py-2** at λ_{em} = 400 nm upon addition of poly rA-poly rU (**pH 5.0**, Na cacodylate buffer, I_c = 0.05 mol dm^{-3}).

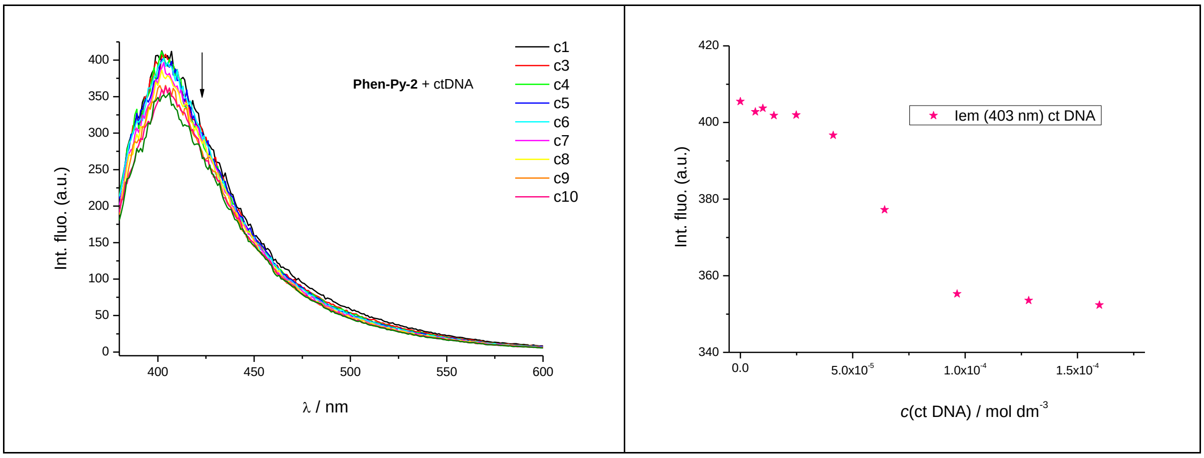

Figure S13. Left: Fluorimetric titration of **Phen-Py-2**, λ_{exc} = 352 nm, $c = 2 \times 10^{-6}$ mol dm^{-3} with *ct*-DNA, Right: Experimental (●) fluorescence intensities of **Phen-Py-2** at λ_{em} = 403 nm upon addition of *ct*-DNA (**pH 7.0**, Na cacodylate buffer, I_c = 0.05 mol dm^{-3}).

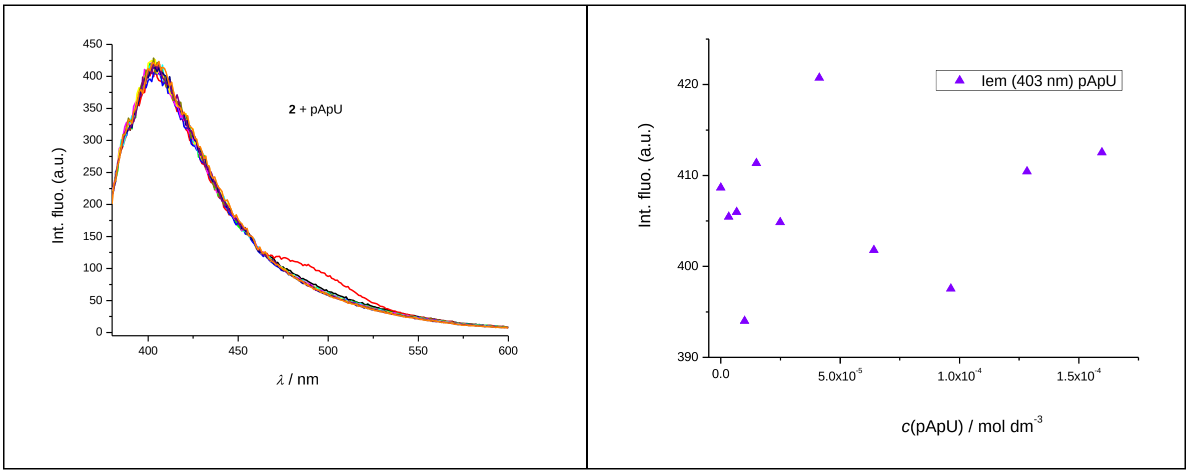

Figure S14. Left: Fluorimetric titration of **Phen-Py-2**, λ_{exc} = 352 nm, *c* = 2 × 10^{-6} mol dm^{-3} with poly rA-poly rU, Right: Experimental (●) fluorescence intensities of **Phen-Py-2** at λ_{em} = 400 nm upon addition of poly rA-poly rU (**pH 7.0**, Na cacodylate buffer, I_c = 0.05 mol dm^{-3}

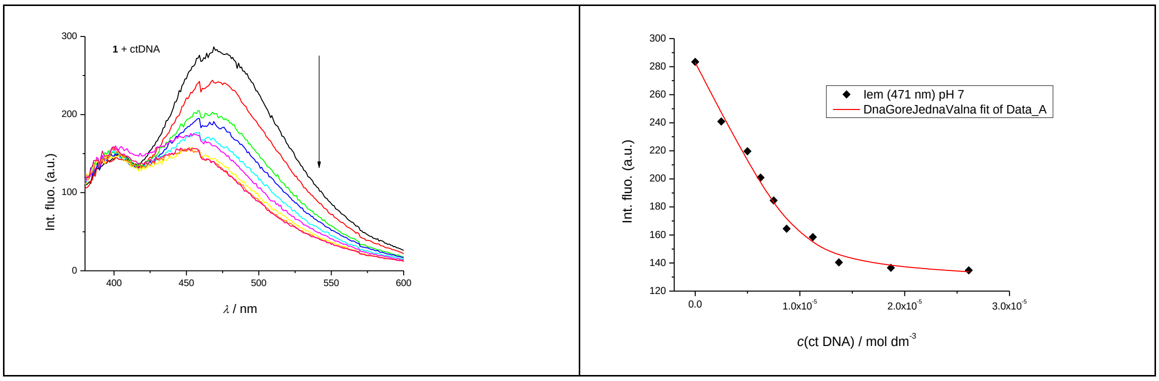

Figure S15. Left: Fluorimetric titration of **Phen-Py-1**, λ_{exc} = 352 nm, *c* = 2 × 10^{-6} mol dm^{-3} with *ct*-DNA, Right: Experimental (●) fluorescence intensities of **Phen-Py-1** at λ_{em} = 471 nm upon addition of *ct*-DNA (**pH 7.0**, Na cacodylate buffer, I_c = 0.05 mol dm^{-3}).

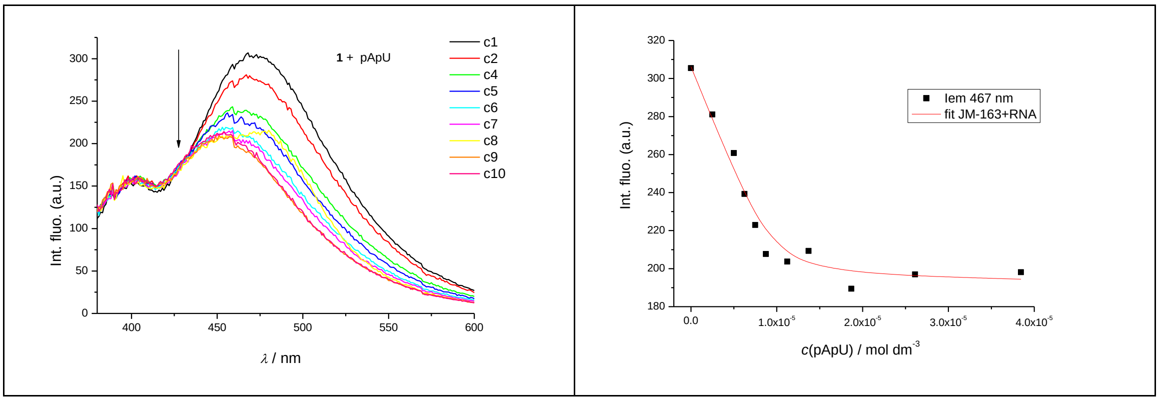

Figure S16. Left: Fluorimetric titration of **Phen-Py-1**, λ_{exc} = 352 nm, *c* = 2 × 10^{-6} mol dm^{-3} with poly rA-poly rU, Right: Experimental (●) fluorescence intensities of **Phen-Py-1** at λ_{em} = 467 nm upon addition of poly rA-poly rU (**pH 7.0**, Na cacodylate buffer, I_c = 0.05 mol dm^{-3}).

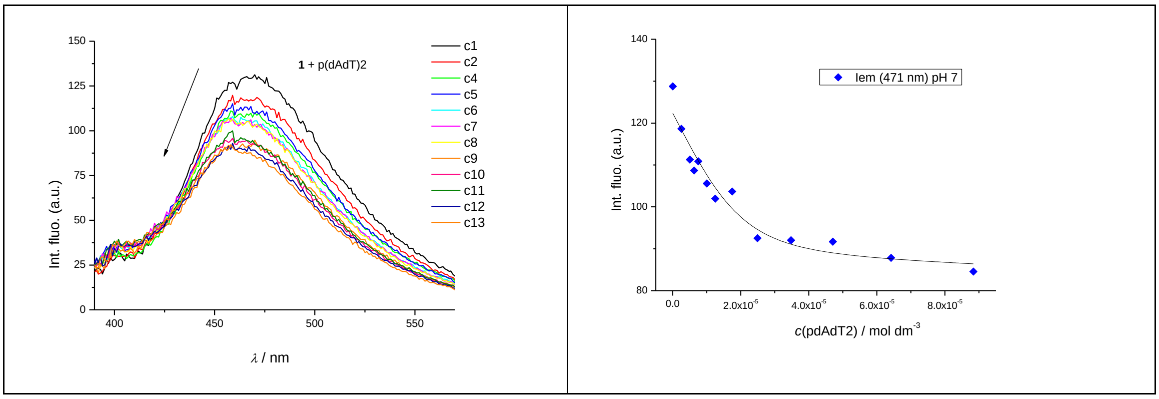

Figure S17. Left: Fluorimetric titration of **Phen-Py-1**, λ_{exc} = 352 nm, c = 2 × 10^{-6} mol dm^{-3} with poly dAdT - poly dAdT, Right: Experimental (●) fluorescence intensities of **Phen-Py-1** at λ_{em} = 471 nm upon addition of poly dAdT - poly dAdT (pH 7.0, Na cacodylate buffer, I_c = 0.05 mol dm^{-3}).

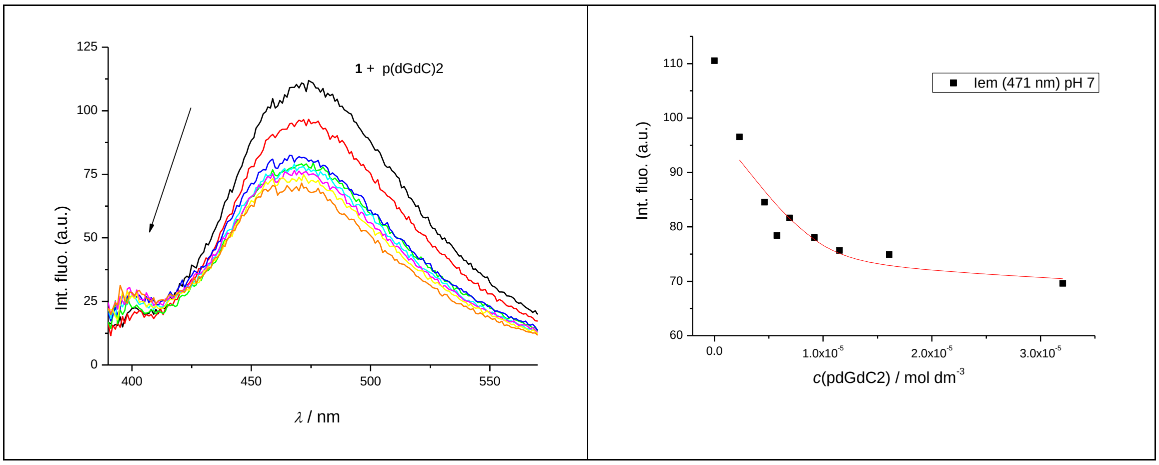

Figure S18. Left: Fluorimetric titration of **Phen-Py-1**, λ_{exc} = 352 nm, c = 2 × 10^{-6} mol dm^{-3} with poly dGdC - poly dGdC, Right: Experimental (●) fluorescence intensities of **Phen-Py-1** at λ_{em} = 471 nm upon addition of poly dGdC - poly dGdC (pH 7.0, Na cacodylate buffer, I_c = 0.05 mol dm^{-3}).

2.3. Circular dichroism (CD) experiments

CD spectroscopy was chosen to monitor conformational changes of polynucleotide secondary structure induced by small molecule binding[2]. Compounds **Phen-Py-1** and **Phen-Py-2** were built using chiral amino acid building blocks and consequently have intrinsic CD spectrum.

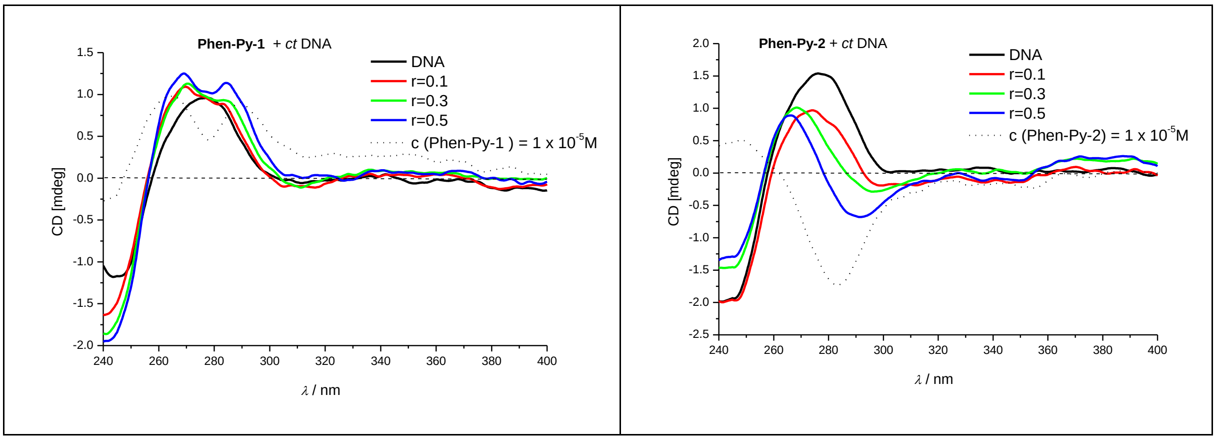

Figure S19. Changes in the CD spectrum of *ct*-DNA (c(DNA) = 2 × 10^{-5} mol dm^{-3}) upon addition of **Phen-Py-1** (left) and **Phen-Py-2** (right) at different molar ratios r = [compound] / [polynucleotide], pH 7.0, sodium cacodylate buffer, I_c = 0.05 mol dm^{-3}

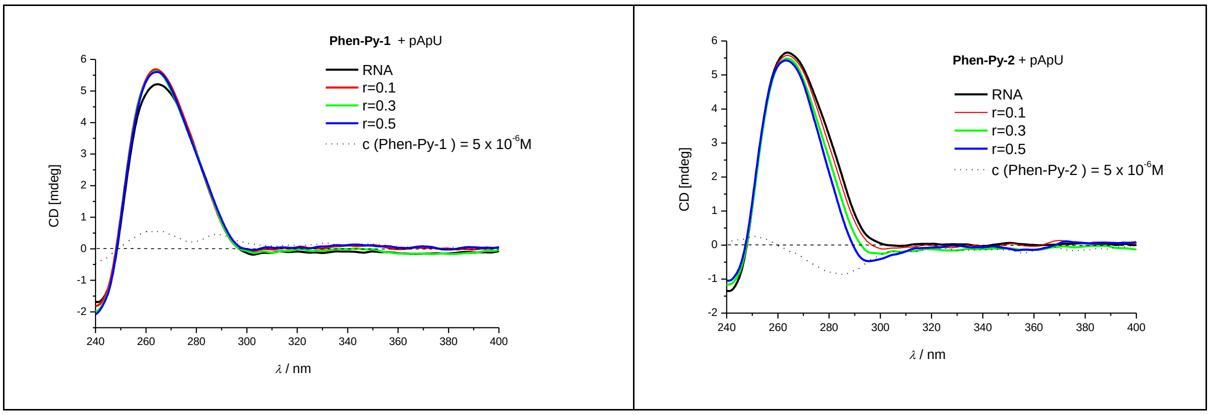

Figure S20. Changes in the CD spectrum of poly rA-poly rU upon addition of **Phen-Py-1** (c(RNA) =1 × 10^{-5} mol dm^{-3}) (left) and **Phen-Py-2;** (c(RNA) = 1 × 10^{-5} mol dm^{-3}) (right) at different molar ratios r = [compound] / [polynucleotide], pH 7.0, sodium cacodylate buffer, I_c = 0.05 mol dm^{-3}

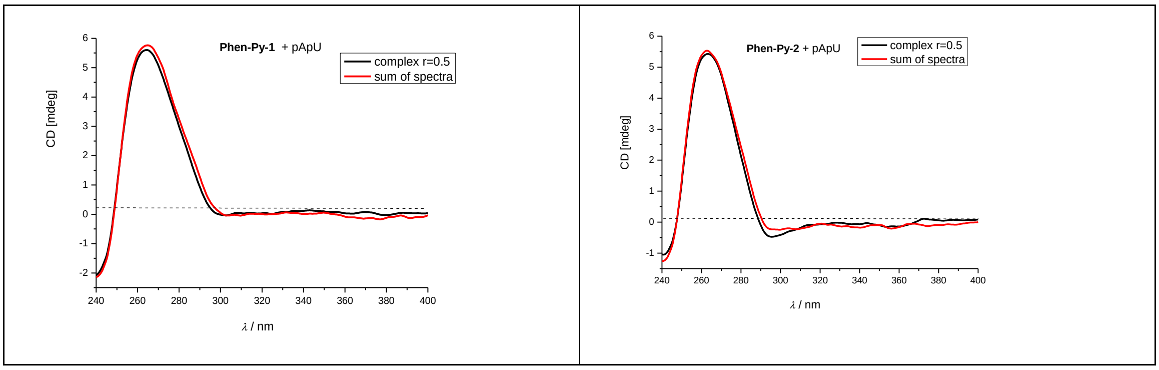

Figure S21. Comparation of spectra of RNA–dye complex (r= 0.5, —) and sum of poly rA-poly rU and dye spectra (—) of appropriate concentrations

3. NMR spectra of **Phen-Py-1** and **Phen-Py-2**

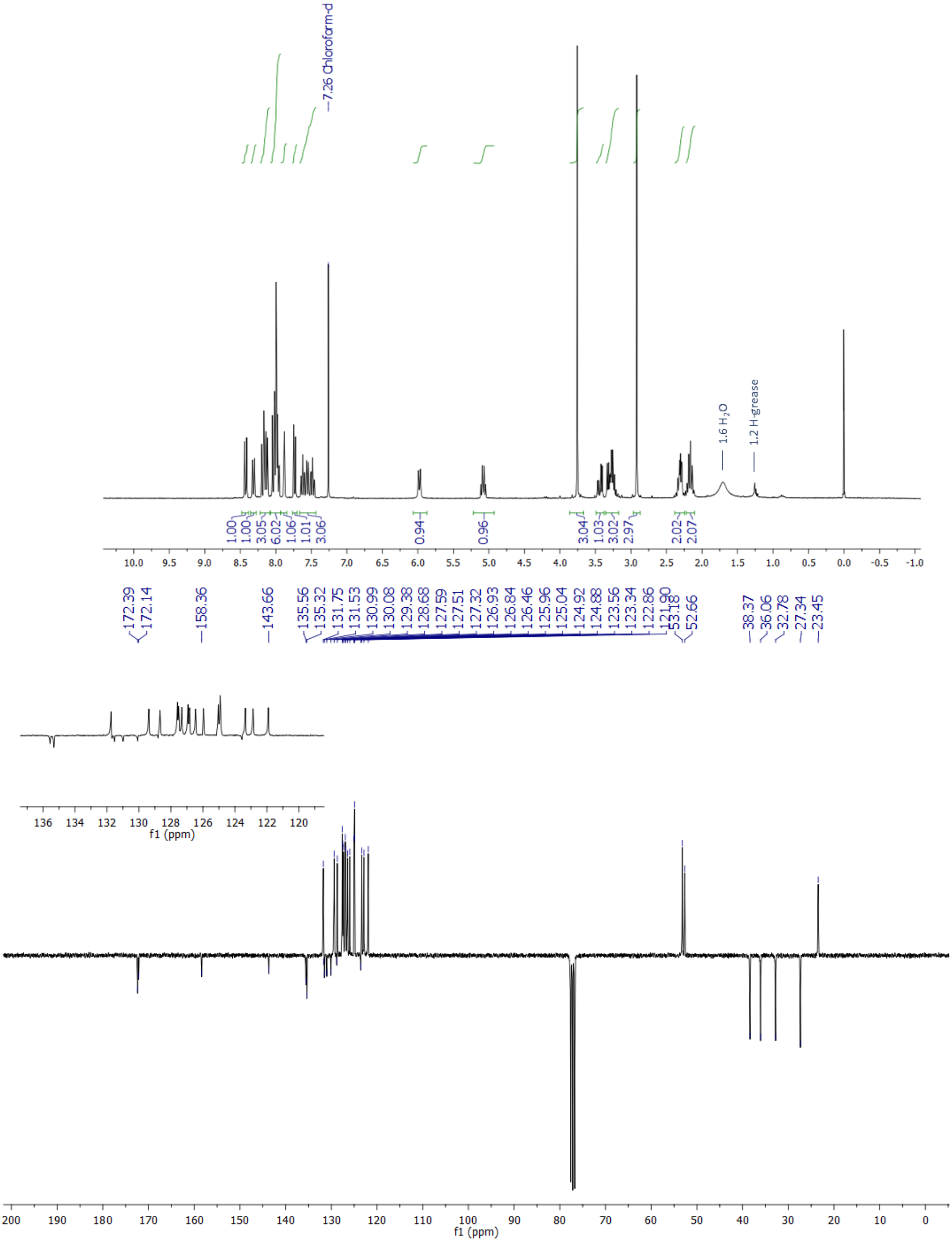

Figure S22. ^1H and ^{13}C NMR (APT) spectra of **Phen-Py-1**.

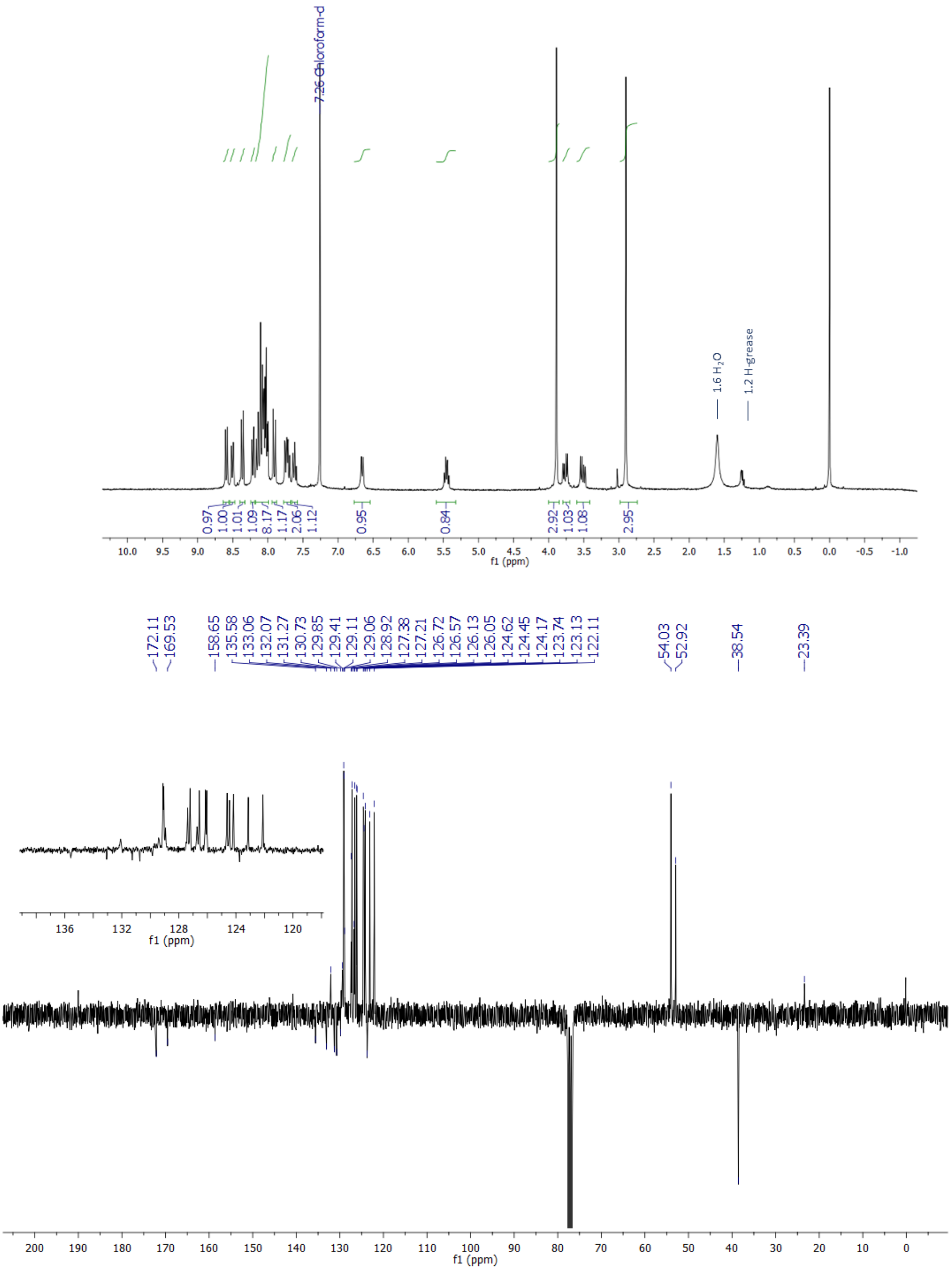

Figure S23. H and ^{13}C (APT) NMR spectra of **Phen-Py-2**.

4. Computational analysis

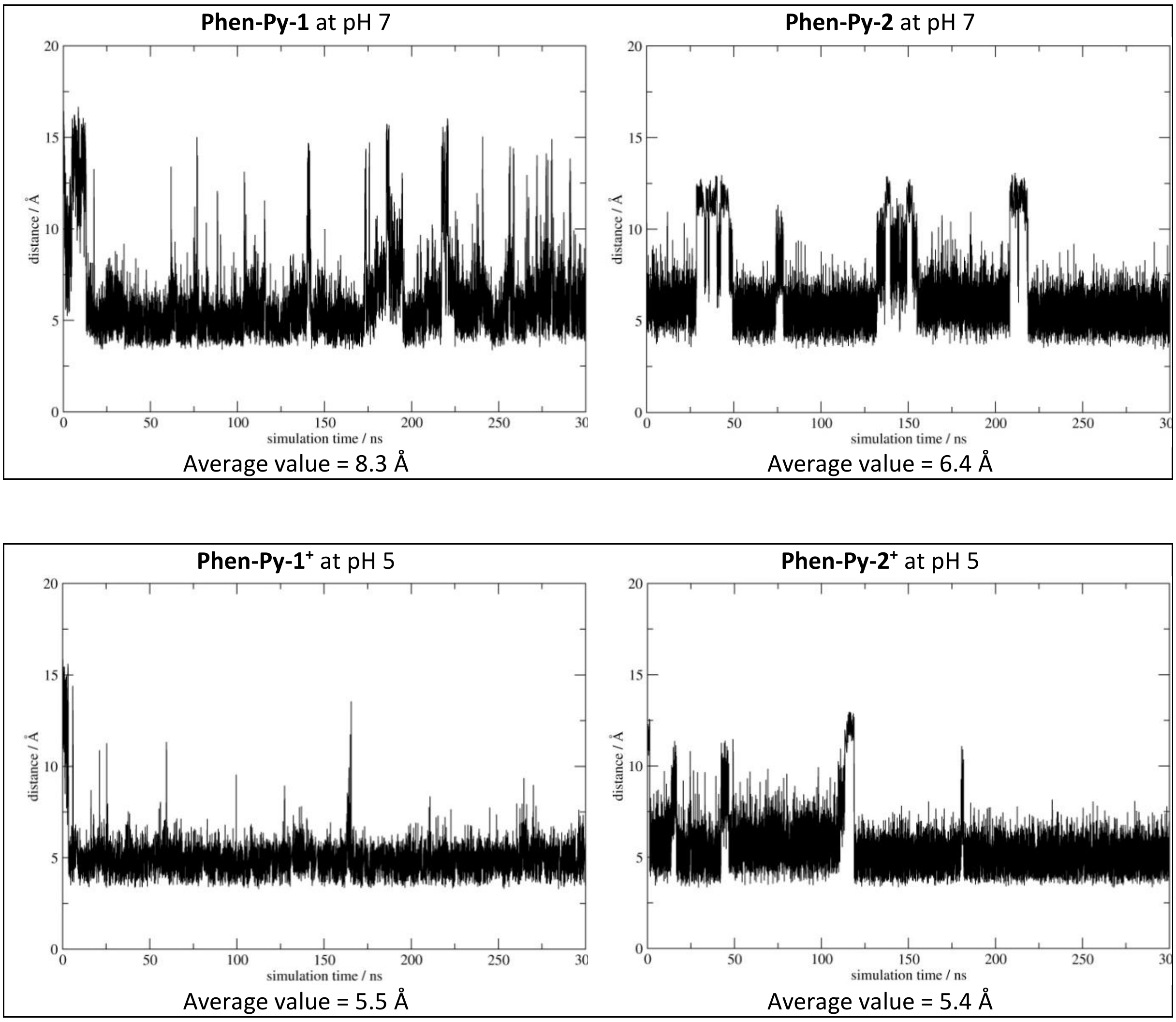

Figure S24. Evolution of distances between the centers of mass among pyrene and phenanthridine aromatic units during 300 ns of MD simulations in **Phen-Py-1** and **-2** conjugates under neutral (pH 7) and acidic (pH 5) conditions.

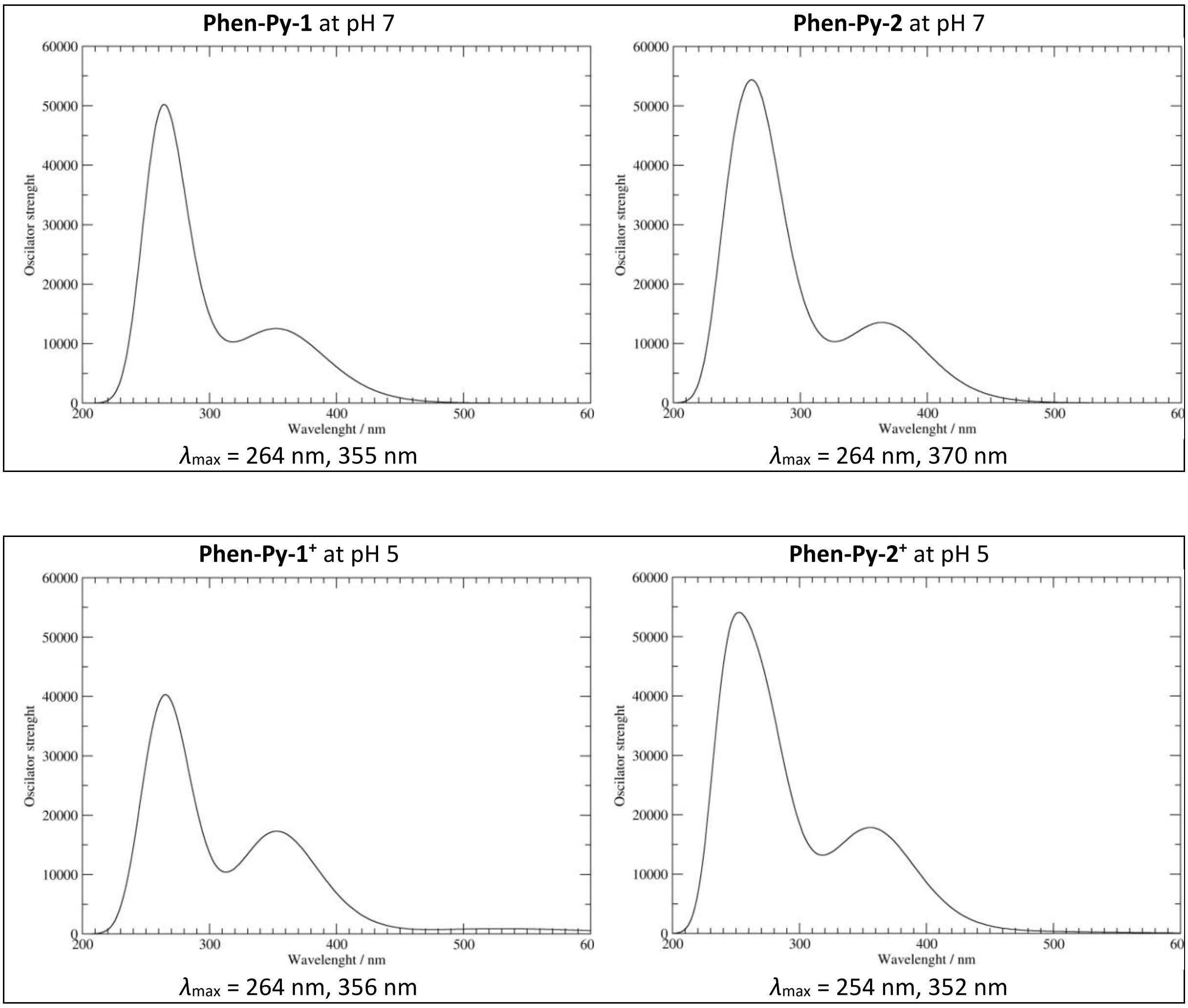

Figure S25. Calculated UV-Vis absorption spectra for **Phen-Py-1-2** conjugates under neutral (pH 7) and acidic (pH 5) conditions using the TD–DFT approach and the (IEF-PCM)/M06–2X/6–31+G(d) level of theory.

5. Confocal microscopy

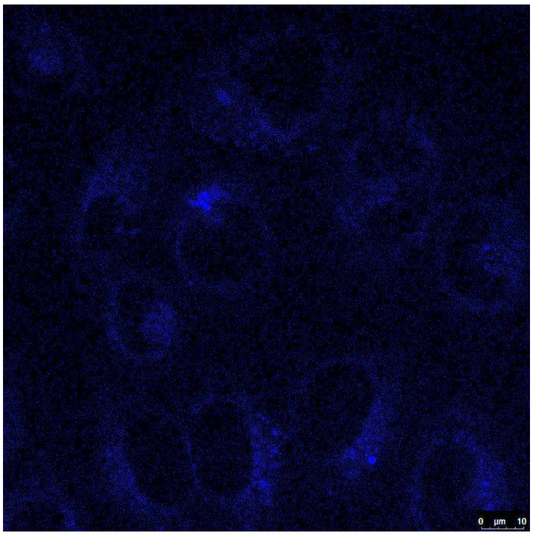

Figure S26. Live-cell image showing accumulation of **Phen-Py-1** dye (blue, 1 µM) in HeLa cells. Cells were treated with 1 µM of the compound and incubated for 60 min in 5% CO_2 at 37 °C. Scale bars, 10 mm. The **Phen-Py-1** dye excitation and emission profiles were previously measured and applied for confocal imaging.

Supporting Information References

[1] Mergny, J. L. and L. Lacroix (2003). "Analysis of thermal melting curves." Oligonucleotides **13**(6): 515-537.

[2] Rodger A., Norden B., In *Circular Dichroism and Linear Dichroism*; Oxford University Press: New York, 1997, Chapter 2.
